# Light Intensity and Temperature Effect on *Salvia yangii* (B. T. Drew) Metabolic Profile *in vitro*

**DOI:** 10.3389/fpls.2022.888509

**Published:** 2022-05-13

**Authors:** Weronika Kozłowska, Adam Matkowski, Sylwia Zielińska

**Affiliations:** ^1^Division of Pharmaceutical Biotechnology, Department of Pharmaceutical Biology and Biotechnology, Wroclaw Medical University, Wroclaw, Poland; ^2^Division of Pharmaceutical Biology and Botany, Department of Pharmaceutical Biology and Biotechnology, Wroclaw Medical University, Wroclaw, Poland

**Keywords:** *Salvia yangii*, light, temperature, carnosic acid, rosmarinic acid, monoterpenes, sesquiterpenes

## Abstract

Plant *in vitro* culture is a feasible system for the testing influence of an environmental factor on the accumulation and chemodiversity of specialized metabolites, especially in medicinal plants. Light and temperature are among the most important factors affecting the physiology of plant organisms but their influence on specific metabolic pathways is not completely understood. Here, we examined the morphogenetic response, photosynthetic pigments content, lipid peroxidation level, DPPH radical scavenging activity, and the production of volatile and non-volatile constituents in *Salvia yangii* B. T. Drew (syn. *Perovskia atriplicifolia* Benth.) *in vitro* cultures kept under different light intensities (70, 130, and 220 μmol m^−2^ s^−1^) and at two selected temperatures (25 and 30°C). The experiment was continued for 7 months to monitor the changes in the treatment response in time. Phytochemical analysis was performed using chromatographic (GC-MS and UHLPC) and spectrophotometric techniques. The light intensity significantly influenced metabolic response in a non-linear manner, whereas temperature-induced adaptive modifications varied within the long cultivation. Significant differences were noted in the content of carnosic and rosmarinic acid, as well as in several sesquiterpenes (alloaromadendrene, β-caryophyllene, α-humulene). At elevated (30°C) temperature, a trend of differently modulated content of two major antioxidants—rosmarinic acid (RA, a phenylpropanoid pathway derived phenolic acid) and carnosic acid (CA, an abietane diterpenoid) was observed, where RA, but not CA, was depending on the light intensity. At 25°C, both compounds depended on light but in various ways. Among the volatile terpenoid compounds, the influence of light was pronounced, leading to modulation of proportions between individual mono- and sesquiterpenes as well as between hydrocarbon and oxygenated compounds. The study provided new information on the metabolic profile plasticity in *S. yangii* and added to the existing knowledge on the chemical adaptations in plant species from severe habitats.

## Introduction

Plant responses to stress caused by intense light and heat have been studied in many species (Ghasemzadeh et al., 2010; Kim et al., 2020; Loi et al., 2021; Rysiak et al., 2021), both for shorter (several hours) and longer (several to several dozen days) periods (Chen et al., 2017; Zhou et al., 2020). Multiple plant defense reactions involved in signal transduction at the cellular and tissue levels have been described (Zdarska et al., 2015; Zandalinas et al., 2020). Apart from the well-examined physiological reactions engaged in the primary metabolism responses against environmental stresses, the production of the so-called plant specialized metabolites, also known as secondary, is still unscrambled (Ibrahim and Jaafar, 2012; Tokarz et al., 2018; Hunt et al., 2021). The latter is of particular importance in the case of chemodiversity and medicinal plant research.

Light intensity and temperature are two main factors affecting photosynthesis and hence determining plant metabolism. According to the Intergovernmental Panel on Climate Change (IPCC, 2021), global temperatures will rise another 1.8–5.7°C by 2100. The constant annual temperature elevation affects the life and functioning of living organisms globally. For obvious reasons, sessile organisms are under the most strain. Concerning the rapidity of warming, plants need to use the already existing evolutionary adaptations and live in a state of continuous adapting to changing conditions at the same time. Plants are a key element of the ecosystem, therefore, our understanding of their adaptation mechanisms at the specialized metabolism level is of great importance for human existence. In this context, the aspects of nutrition, pharmaceutics, ecological heritage, and loss of biodiversity are the most important.

In nature, plants are exposed to many stresses including light, temperature, water limitations, and deficiencies/excess of minerals. Plant response to environmental factors is multidimensional to ensure survival. Within minutes, chloroplast movement regulates light absorption on a cellular level (Ruban, 2009). Also, minutes are needed to propagate the received signal from a local tissue to an entire plant by electric, calcium, reactive oxygen species (ROS), or hydraulic waves. The hormonal and metabolic response is initiated in parallel. Depending on the intensity and duration of the signal, plants initiate mechanisms involving gene expression and the regulation pass on transcriptional, translational, and post-translational levels (Kollist et al., 2019; Zandalinas et al., 2020). This starts within hours and involves metabolic or/and developmental mechanisms. The first occurs within hours–days, and the second is prolonged to days–weeks (Ruban, 2009).

The impact of high irradiance and temperature on the phenolic accumulation in different plant species has been extensively studied (Chen et al., 2017; Raffo et al., 2019; Yeddes et al., 2019), and stress-induced metabolic fluctuations were observed (Cramer et al., 2011). An accumulation of metabolites has been often detected as a response to biotic or abiotic stress. However, most observations were made in short-term studies (Sun et al., 2017; Yang et al., 2018; Yeddes et al., 2019), while the long-term effect of selected factors such as light intensity or elevated temperature on plant reactions needs further research. In that respect, the experiments mimicking global warming effect would be of the highest interest.

*Salvia yangii* (B. T. Drew) is one of the plants that can provide a model of the dynamic changes in the specialized metabolites composition under the influence of elevated temperature and light. It is a subshrub native to central-west Asia, with a range of natural occurrences comprising Iran, Pakistan, Afghanistan, and west China. Its natural habitats require a resistance both to intense solar irradiation and large temperature fluctuations.

In 2017, phylogenetic studies moved this taxon that was previously categorized as *Perovskia atriplicifolia* (Benth.) to *Salvia* genus. It was renamed *S. yangii* (Drew et al., 2017). The plant occurs in arid ecosystems and can withstand drought but requires at least seasonal soil humidity. *S. yangii* is a source of specialized nor-abietane derivatives occurring only in a few species within the Lamiaceae family (Birtić et al., 2015), which were united into the extended *Salvia* genus. The aerial parts of plants from previous *Perovskia* subgenus produce high amounts of volatile terpenoid compounds (Pourmortazavi et al., 2003; Erdemgil et al., 2007). Unique metabolic profile, natural flexibility, and resistance to changing environmental factors prompted us to study multidimensional stress response in this intriguing plant species.

In the present study, we monitored long-lasting metabolic adaptations rather than a rapid response to stress factors. The *in vitro* experiments were performed on the species that naturally occur in highly insolated and hot areas, but with large temperature fluctuations between day and night. The long-term effects of two temperature regimes and three light intensities in *S. yangii in vitro* shoot cultures were investigated. The content of photosynthetic pigments, as well as volatile and non-volatile specialized metabolites, was estimated using chromatographic and spectrophotometric techniques. Lipid peroxidation level marked plant redox balance, whereas DPPH was used to monitor radical scavenging properties.

## Materials and Methods

### Plant Material

Shoot cultures of *S. yangii* were induced from aseptically germinated seedlings. Seeds were collected from plants cultivated in experimental plots in a full sun exposition in the Botanical Garden of Medicinal Plants at the Wroclaw Medical University (GPS 51.1172228, 17.0747974) in 2017. A plant voucher specimen was deposited in the herbarium of the Botanical Garden of Medical Plants at the Wroclaw Medical University, reference number P-123.

The seeds were surface sterilized with 5% sodium hypochlorite (23 min), rinsed three times (5 min) with sterile water, and planted on a sterile MS (Murashige and Skoog, 1962) culture medium. MS medium supplemented with sucrose (3%) and agar (0.6%) (LAB-AGAR, Biocorp, Poland), was used during all experiments. The pH was adjusted to 5.8 before autoclaving (121°C, 20 min). Shoot tips (1 cm long) of aseptically grown seedlings were used as explants. Four explants were placed in each round-bottom culture jar (WECK Rundrand-glass 60, J. Weck & Co, Germany) containing 100 ml of culture medium. Shoots were cut and transferred into fresh media every 49 days.

The cultures were maintained in the growth chamber (Conviron, A1000, Canada) under a 16/8-h (light/dark) photoperiod with cool fluorescent light (LUMILUX, HE21W/840 lamps, OSRAM, Italy). Plants were cultured in three different light intensity regimes 70 μmol m^−2^ s^−1^ (low), 130 μmol m^−2^ s^−1^ (moderate), and 220 μmol m^−2^ s^−1^ (high) and at two different temperature regimes 25 ± 2°C/20 ± 2°C (light/dark) and 30 ± 2°C/25 ± 2°C (light/dark) for 49, 98, 147, and 196 days. Data on the number of axillary shoots per explant, axillary shoot length, number of nodes per explant (axillary shoot), and plant weight were recorded every 49 days of culture. A total of 24 explants were used for each experimental treatment. The plant material was chemically characterized using chromatographic techniques.

### Solvents and Reference Substances

Methanol and ethyl acetate were purchased from Chempur, Poland. Acetonitrile and formic acid were from Sigma-Aldrich (Steinheim, Germany). Cyclohexane was from Merck (Burlington, MA, United States). Authentic standards of RA and CA were obtained from Sigma-Aldrich. The Folin-Ciocalteau reagent was from POCh (Gliwice, Poland).

### Photosynthetic Pigments Estimation

The photosynthetic pigments were estimated according to the modified method by Lichtenthaler and Buschmann (2001). Fresh plant material (200 mg) was homogenized using mortar and pestle, and extracted with 95% ethanol (5 ml, v/v) and a pinch of calcium carbonate (CaCO_3_). Samples were centrifuged for 15 min at 5,000 rpm. The supernatant was collected, diluted (1:6 in 95% EtOH), and the absorbance was measured at 470, 649, and 664 nm using a microplate spectrophotometer (μQuant, Biotek, United States). Chlorophylls (Chl a, b) and carotenoid content were calculated according to Lichtenthaler and Buschmann (2001).

### Lipid Peroxidation Status of Plant Tissue

Lipid peroxidation was estimated by measuring the formation of malonyldialdehyde (MDA) content with 2-thiobarbituric (TBA) acid according to the modified method of Tokarz et al. (2018). Micronized samples (10 mg DW) were sonicated in 0.1% trichloroacetic acid (TCA) for 15 min and centrifuged for 10 min at 13,000 x g. The supernatant was mixed with 20% TCA containing 0.5% TBA. The reaction was incubated at 95°C for 15 min and immediately stopped by transferring on ice. The absorbance was measured at 450, 532, and 600 nm (BioTeq, μQuant™, Microplate Spectrophotometer). The content of MDA as thiobarbituric acid reactive substances (TBARS) was calculated based on the following formula: (μM MDA/g DW) = 6.45 (OD_532_ – OD_600_) – 0.56 OD_450_.

### Total Phenolic Content

The Folin-Ciocalteu test was used to determine the total content of reducing phenolics. The plant material (10 mg DW) was extracted with 80% MeOH (1 ml, v/v) in an ultrasonic bath for 15 min. Samples were centrifuged for 10 min at 13,000 g. The obtained supernatant was diluted and Folin-Ciocalteu reagent (100 μl) was added followed by 5% Na_2_CO_3_ (1.6 ml, w/v). Samples were incubated for 15 min at 40°C and centrifuged for 20 min at 4,000 g. The absorbance was measured at 740 nm and chlorogenic acid was used as a reference substance for the calibration curve. Calculations were based on the regression equation and the obtained results were expressed as milligram chlorogenic acid equivalents per 1 g of plant tissue dry weight.

### DPPH Assay

The assay was performed according to a modified method of Brand-Williams et al. (1995). Samples of dried *S. yangii in vitro* shoots (10 mg) were extracted with 1 ml of 80% MeOH (v/v). The obtained extracts were further diluted to relative concentrations 0.04; 0.08; 0.16; 0.24; 0.32; 0.48; 0.64; 0.8 (v/v) and filled with equal part of 0.2 mM methanolic DPPH^∙^ solution. The absorbance was read at 517 nm immediately after adding and after 1, 5, and 30 min. The reaction mixture was incubated in the dark at room temperature; 80% MeOH mixed with the DPPH solution was used as a control. The percentage of the scavenging effect was calculated from the following equation:


$$\begin{array}{ll} \%\;scavenging= & (Abs_{DPPH}-(Abs_{sample}-Abs_{control}))/Abs_{DPPH}\\ \; & \times 100, \end{array}$$

where Abs_DPPH_ – absorbance of DPPH with MeOH; Abs_sample_ absorbance of the sample with DPPH; Abs_control_- absorbance of the sample without DPPH.

From the obtained results, the EC_50_ value was calculated using AAT Bioquest, Inc. (2021).

### Non-volatile Compounds Extraction

The plant material stored at −20°C was lyophilized and used for a two-step extraction. Samples (50 mg) were extracted with ethyl acetate (EtOAc) (2.5 ml), sonicated for 15 min, and centrifuged for 5 min at 5,000 rpm. The procedure was performed twice and the supernatants were combined. After extraction with EtOAc, the sediment of the plant material was extracted with 80% methanol (MeOH) (2.5 ml), repeating the previous procedure. Both extracts, EtOAc and MeOH, were evaporated to dryness under N_2_, dissolved in 1 ml of 80% MeOH, filtered with 0.22 μm PTFE syringe filters, and used for the UHPLC analysis.

### UHPLC Analysis

The UHPLC-DAD analysis was performed using Thermo Ultimate 3000 RS (Thermo-Fisher Scientific, Waltham, MA) chromatographic system coupled with PDA/diode array detector. Separation was performed on Kinetex C18 column (2.1 mm × 100 mm, 2.6 μm, Phenomenex, United States). The mobile phase consisted of A−0.1% (v/v) formic acid in water; B−0.1% (v/v) formic acid in acetonitrile using a gradient program: 0–10 min 5–20%; 10–23 min 20–95%; 23–25 min 95%; 25–28 min 95–5%, 28–31 min 5% of B. Flow rate was 0.3 ml/min and the column was maintained at 25°C (±1°C). Detection was set at 320 nm for RA and 280 nm for CA. Identification of compounds was based on the comparison of the actual retention time of authentic reference standards (Sigma-Aldrich). The data were acquired and processed using Chromeleon 6.8 software (Thermo Scientific).

### VOCs Solvent Extraction

The plant material collected from each experimental treatment was stored at −20°C. Before the phytochemical analysis, it was freeze-dried and homogenized, and three replicates of the representative samples were prepared. Accurately weighted plant material (100 mg) was extracted with cyclohexane (1 ml). Samples were vortexed, sonicated for 15 min, and left for 24 h at 4°C. The obtained extract was centrifuged (7,000 rpm, 5 min), filtered using 0.22 μm PTFE syringe filters, and immediately analyzed with GC-MS.

### Gas Chromatography-Mass Spectrometry (GC-MS)

Gas chromatography was performed using Agilent 7890B GC system coupled with Agilent 7000GC/TQ and PAL RSI85 autosampler (Agilent Technologies, Palo Alto, CA). The column used in the analysis was HP-5 MS; 30 m × 0.25 mm × 0.25 μm (Agilent Technologies, Palo Alto, CA). Temperature program: 50°C up to 130°C at 4°C min^−1^, then up to 270°C at 10°C min^−1^, and hold isothermal for 2 min. The injector temperature was set to 250°C. Helium was used as a carrier gas at a constant flow of 1 ml/min, the split ratio was set to 1:100. Mass spectrometer settings: ionization voltage 70 eV, the temperature of transfer line, source, and quadrupole was 320, 230, and 150°C, respectively. Detection was performed at a full scan mode over the mass range of 30–400 amu. Identification was based on the comparison of the retention index (RI) and mass spectra with NIST 17.1 library and literature data (Adams, 2007). Linear retention indexes were determined by analyzing a mixture of C8–C20 saturated alkanes standard (Sigma-Aldrich), in the same conditions as used for VOCs samples. To evaluate the relative abundance of individual constituents (expressed as percentages of total volatile organic compounds) peak area normalization measurement was carried out. Samples were prepared in triplicate for each experimental treatment.

### Statistical Analyses

Statistical analyses were performed using Statistica 12.0 (StatSoft Inc., Poland). To establish the differences between samples, one-way ANOVA followed by the Kruskal–Wallis test were performed. Statistical significance was set at *p* < 0.05. Factorial analysis based on PCA was performed for selected VOCs to understand the relationships between individual compounds. The relative contribution analysis was performed to show the effect of light, temperature, and culture duration as well as interactions between them on morphogenetic and metabolic responses.

## Results

### Shoot Cultures

**Figure 1:**
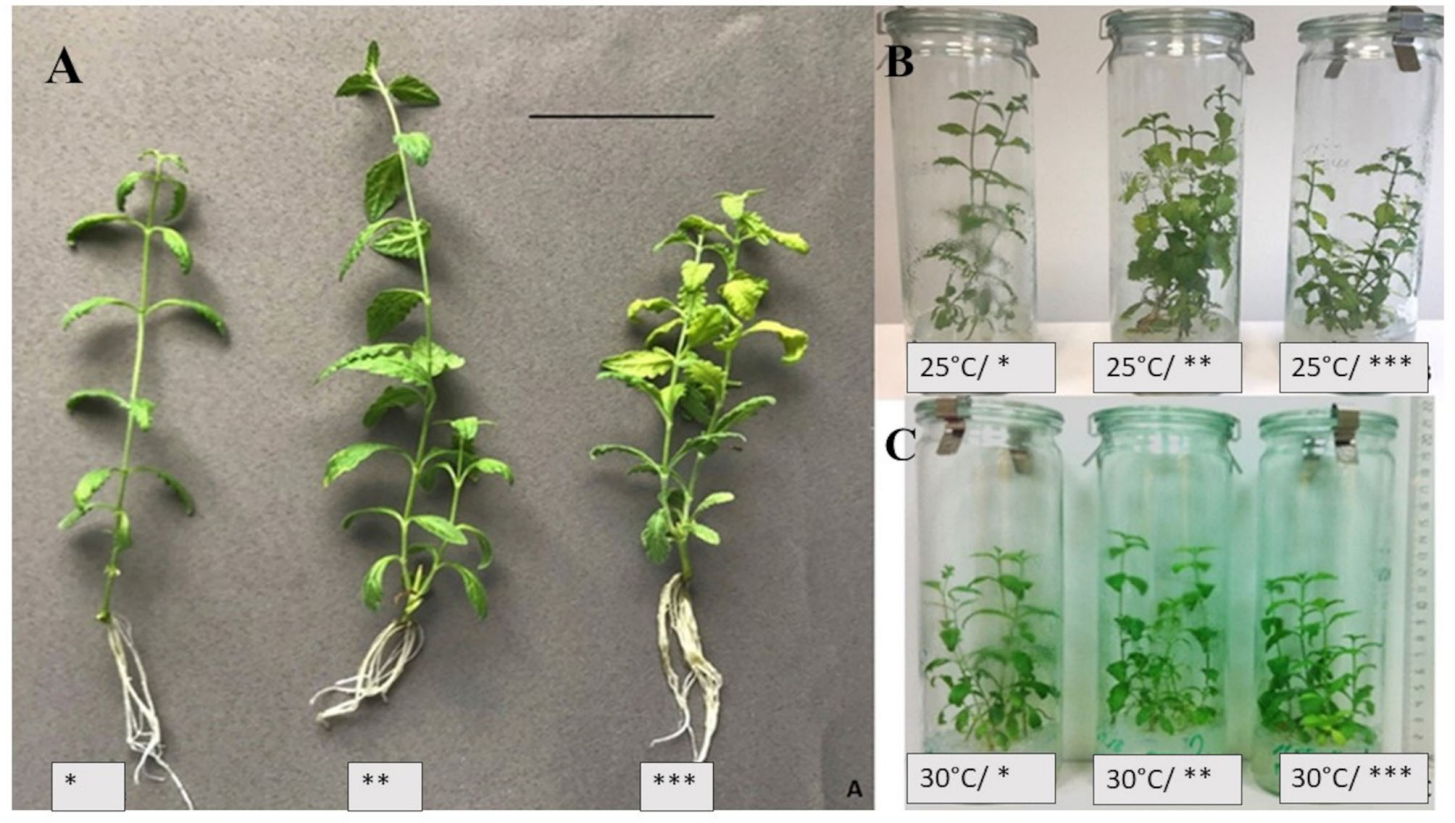
*S. yangii in vitro* shoots cultured under three cool fluorescent white light intensities at 25°C **(A)**; and at two temperatures: 25°C **(B)** or 30°C **(C)**; LI: *70 μmol m^−2^ s^−1^; **130 μmol m^−2^ s^−1^; ***220 μmol m^−2^ s^−1^; scale bar: 5 cm.

The light intensity and temperature influenced all measured features during all observation periods (Table 1, Figure 1). The shoots remained vigorous and did not show morphological abnormalities during all experimental treatments.

**Table 1:** Morphogenetic response of *S. yangii in vitro* shoots cultured under three cool fluorescent light intensities (LI): *70 μmol m^−2^ s^−1^; **130 μmol m^−2^ s^−1^; ***220 μmol m^−2^ s^−1^ in two temperature regimes (T): 25°C and 30°C, culture duration (CD): 49, 98, 147, 196 days.

**T**	**CD**	**LI**	**Mean number of axillary shoots per explant**	**% of explants developing two axillary shoots**	**Mean length of axillary shoots (cm)**	**Mean number of nodes/cm of axillary shoot**	**Mean mass of shoot (g)**	**Callus formation (%)**	**Mean roots length (cm)**
25°C	49	*	1.32 ± 0.48^aA#^	31.58 ± 0.48^aA#^	12.71 ± 5.78^aA#^	0.78 ± 0.20^aA#^	0.18 ± 0.12^aA#^	0.00 ± 0.00^aA#^	1.72 ± 0.73^aA#^
		**	1.70 ± 0.47^aA#^	70.00 ± 0.47^aA#^	13.10 ± 3.42^aA#^	0.91 ± 0.19^abA#^	0.18 ± 0.04^abA#^	0.00 ± 0.00^aA#^	1.51 ± 0.52^aA#^
		***	1.45 ± 0.51^aA#^	45.00 ± 0.5^aA#^	10.29 ± 5.43^aA#^	1.17 ± 0.23^bA#^	0.40 ± 0.20^bA#^	0.00 ± 0.00^aA#^	2.00 ± 1.00^aA#^
	98	*	1.29 ± 0.46^aA#^	28.57 ± 0.46^aA#^	13.17 ± 5.27^aA#^	0.74 ± 0.21^aA#^	0.20 ± 0.07^aA#^	0.00 ± 0.00^aA#^	2.50 ± 0.60^aA#^
		**	1.39 ± 0.50^aA#^	38.89 ± 0.50^aA#^	15.27 ± 3.97^aA#^	0.84 ± 0.09^abA#^	0.33 ± 0.14^aAC#^	8.00 ± 1.20^bB#^	5.30 ± 0.40^bB#^
		***	1.65 ± 0.48^aA#^	65.00 ± 0.48^aA#^	15.37 ± 6.01^aA#^	0.96 ± 0.23^bA#^	0.39 ± 0.11^aA#^	0.00 ± 0.00^aA#^	3.20 ± 0.32^aA#^
	147	*	1.48 ± 0.54^aA#^	54.16 ± 0.49^aA#^	12.97 ± 6.72^aA#^	0.68 ± 0.22^aA#^	0.36 ± 0.18^aA#^	0.00 ± 0.00^aA#^	6.71 ± 0.62^aA#^
		**	1.14 ± 0.39^aA#^	19.45 ± 0.38^aA#^	13.27 ± 4.13^aA#^	0.70 ± 0.17^aB#^	0.42 ± 0.21^aBC#^	0.00 ± 0.00^aA#^	5.53 ± 1.02^aB#^
		***	1.62 ± 0.53^aA#^	67.44 ± 0.45^aA#^	9.43 ± 3.99^aA#^	0.99 ± 0.37^bA#^	0.47 ± 0.41^aA#^	6.00 ± 0.60^bB#^	5.69 ± 0.74^aA#^
	196	*	1.50 ± 0.54^aA#^	55.70 ± 0.48^aA#^	12.45 ± 5.24^aA#^	0.73 ± 0.24^aA#^	0.31 ± 0.14^aA#^	0.00 ± 0.00^aA#^	6.00 ± 0.36^aA#^
		**	1.36 ± 0.53^aA#^	40.80 ± 0.48^aA#^	13.16 ± 5.23^aA#^	0.82 ± 0.24^aAB#^	0.69 ± 0.26^bB#^	0.00 ± 0.00^aA#^	4.78 ± 0.56^aB#^
		***	1.73 ± 0.48^aA#^	78.48 ± 0.39^aA#^	9.84 ± 5.93^aA#^	1.28 ± 0.43^bA#^	0.34 ± 0.23^aA#^	0.00 ± 0.00^aA#^	5.23 ± 0.36^aA#^
30°C	49	*	1.47 ± 0.52^aA#^	46.67 ± 0.52^aA#^	15.21 ± 6.45^aA#^	0.69 ± 0.18^aA#^	0.36 ± 0.14^aA##^	0.00 ± 0.00^aA#^	1.06 ± 0.12^aA#^
		**	1.82 ± 0.39^bA##^	82.32 ± 0.39^aAB#^	21.82 ± 5.47^bA##^	0.69 ± 0.09^aA##^	0.52 ± 0.22^aA##^	8.00 ± 0.50^bB##^	1.92 ± 0.40^bA##^
		***	1.77 ± 0.44^aA#^	76.92 ± 0.44^aA#^	13.16 ± 6.42^aA#^	1.23 ± 0.24^bA#^	0.60 ± 0.28^aA##^	0.00 ± 0.00^aA#^	2.20 ± 0.22^aA#^
	98	*	1.70 ± 0.47^aA#^	70.00 ± 0.47^aA#^	14.56 ± 4.35^aA#^	0.76 ± 0.11^aAB#^	0.27 ± 0.12^aA#^	0.00 ± 0.00^aA#^	3.40 ± 0.40^aA#^
		**	1.37 ± 0.49^aB#^	36.84 ± 0.49^bA#^	14.63 ± 3.05^aB#^	0.71 ± 0.10^aA##^	0.53 ± 0.17^bA##^	0.00 ± 0.00^aA##^	6.50 ± 0.52^aB#^
		***	1.90 ± 0.31^aA##^	89.47 ± 0.32^abA#^	8.21 ± 3.50^aA##^	1.47 ± 0.50^bA##^	0.26 ± 0.13^bA#^	11.00 ± 0.50^bB##^	5.20 ± 0.71^aA##^
	147	*	1.89 ± 0.31^aA#^	89.47 ± 0.32^aA#^	11.67 ± 4.12^aA#^	0.98 ± 0.28^aB#^	0.22 ± 0.11^aA#^	0.00 ± 0.00^aA#^	4.82 ± 0.43^aA#^
		**	1.90 ± 0.31^bA##^	90.00 ± 0.31^aB##^	19.16 ± 4.66^bA##^	0.77 ± 0.13^bA#^	0.66 ± 0.18^bA#^	0.00 ± 0.00^aA#^	6.32 ± 1.00^bA##^
		***	2.00 ± 0.00^aA#^	100.00 ± 0.00^aA#^	11.80 ± 3.50^aA#^	1.17 ± 0.21^cA#^	0.49 ± 0.18^bA#^	0.00 ± 0.00^aA#^	6.80 ± 0.38^aA#^
	196	*	1.65 ± 0.49^aA#^	65.00 ± 0.68^aA#^	11.82 ± 6.34^aA#^	0.89 ± 0.31^aAB##^	0.29 ± 0.16^aA#^	0.00 ± 0.00^aA#^	4.90 ± 0.63^aA#^
		**	1.80 ± 0.41^bA##^	80.00 ± 0.4^aAB#^	20.21 ± 4.36^bA##^	0.68 ± 0.14^aA##^	0.64 ± 0.16^bA#^	0.00 ± 0.00^aA#^	4.12 ± 0.35^bA##^
		***	2.00 ± 0.00^aA#^	100.00 ± 0.00^aA#^	10.98 ± 4.06^aA#^	1.24 ± 0.25^bA#^	0.43 ± 0.21^aA#^	0.00 ± 0.00^aA#^	5.22 ± 0.32^aA#^

*Different letters indicate significant differences (p < 0.05) according to the Kruskal–Wallis test; small letters—differences between samples from three LI; capital letters—differences between samples from four culture durations measured in days, #,##—differences between samples derived from two temperature regimes within a single culture duration period*.

The shoots grown in lower LI developed thinner stems and smaller leaf blades (data not shown). However, the stems were more hydrated and the leaf blades were larger when higher LI was used, although, in 220 μmol m^−2^ s^−1^, they were lighter green with chlorophyll-less sectors (Figure 1).

The shoots obtained from nodal segments developed 1 or 2 axillary shoots depending on culture conditions. Explants that developed two shoots were observed more often when higher light intensity and higher temperature were used (Table 1).

The number of nodes per 1 cm of the axillary shoot was the highest in 220 μmol m^−2^ s^−1^ LI under both temperature treatments. Additionally, shoots cultured in 130 μmol m^−2^ s^−1^ at 30°C were longer with the lowest number of nodes per cm of the stem (Table 1). This was also reflected in biomass production which was the highest at this light treatment (data not shown). All explants were rhizogenic, whereas callus formation was observed only in the case of several explants with no correspondence to the light or temperature treatment (Table 1).

The relative contribution analysis showed that the length of axillary shoots, number of nodes/cm of the axillary shoot, and mass of the shoot were significantly influenced by the LI. In turn, the mean number of axillary shoots was affected mainly by temperature, whereas root length by the culture duration (Supplementary Table 3).

### Photosynthetic Pigments

**Figure 2:**
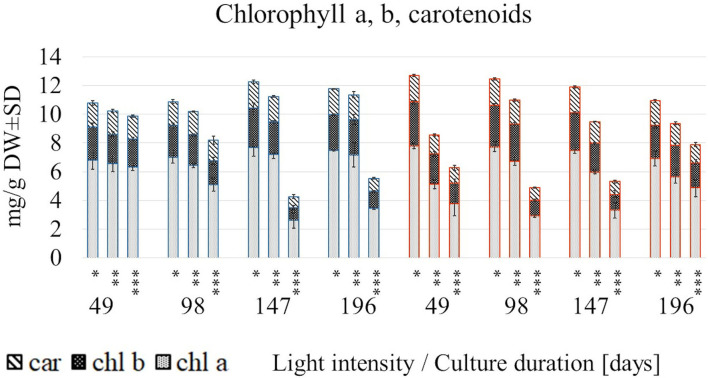
The content of chlorophyll a, b and carotenoids in *S. yangii* shoots cultured in different LI and temperature regimes; *70 μmol m^−2^ s^−1^; **130 μmol m^−2^ s^−1^; ***220 μmol m^−2^ s^−1^; blue: 25°C, red: 30°C.

The photosynthetic pigments were produced in a light intensity-dependent manner, although reacted differentially in both temperatures (Figure 2, Supplementary Table 1).

A rapid decrease in their content under 220 μmol m^−2^ s^−1^ at 30°C occurred earlier than at 25°C (after 49 and 147 days, respectively). Conversely, at 25°C in lower and moderate light intensities, the photosynthetic pigments increased after 147/196 days, whereas in high light decreased almost twice. Pigments content at a low light intensity remained relatively stable throughout the culture, disregarding temperature. In turn, the rising temperature caused a significant loss of each pigment content under moderate and high light being the lowest at 49 days. However, the trend was reversed after a longer culture (147 and 196 days) at 30°C (Figure 2).

**Figure 3:**
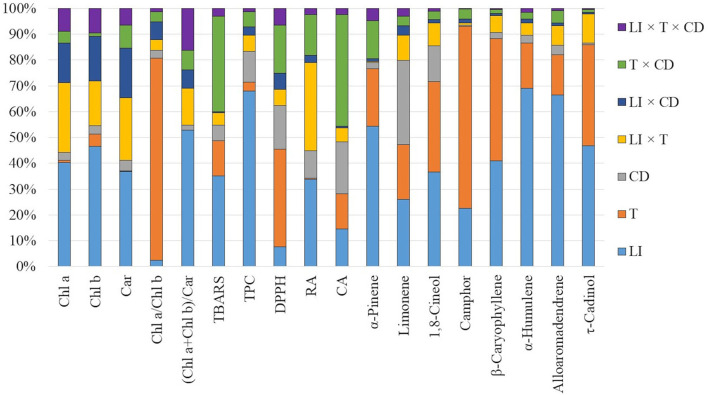
The relative contribution of light intensity (LI), temperature (T), and culture duration (CD) and their interactions to variation in the metabolic response of *S. yangii in vitro* shoots.

In all examined regimes, the total chlorophylls to total carotenoids ratio was inversely related to LI, whereas the chlorophyll a/b ratio in most cases was the lowest in 130 μmol m^−2^ s^−1^ (Supplementary Table 1). The relative contribution analysis showed that mainly light influenced the chl a, chl b, and car content, as well as the (chl a + chl b)/car ratio. In turn, chl a/ chl b ratio was more affected by the temperature treatment (Figure 3, Supplementary Table 5).

### Lipid Peroxidation Status of Plant Tissue

Thiobarbituric acid reactive substances (TBARS) accumulated with culture duration at 25°C under two higher light intensities. At 30°C, the TBARS content was highest after 49 days, and during the culture, dropped down to levels comparable to the initial values at 25°C. However, at both temperatures, TBARS were higher under moderate and high light (Table 2, Figure 3).

**Table 2:** TBARS accumulation in *S. yangii* shoots cultured in different light intensities (LI) and temperature regimes.

**LI/CD**	**25** **°** **C**				**30** **°** **C**			
	**49**	**98**	**147**	**196**	**49**	**98**	**147**	**196**
^*^	24.10 ± 2.41^aA#^	20.30 ± 2.85^aA#^	25.52 ± 2.76^aAB#^	28.88 ± 2.76^aB#^	34.64 ± 4.44^aA#^	26.20 ± 1.05^aB#^	19.35 ± 1.61^aB#^	21.38 ± 3.52^aAB#^
^**^	27.76 ± 4.17^bA#^	31.79 ± 2.26^bAB##^	28.07 ± 2.84^aA#^	39.30 ± 2.76^bB#^	40.71 ± 3.08^abA#^	28.97 ± 1.49^abB##^	29.93 ± 2.53^abABC#^	24.81 ± 1.47^aBC#^
^***^	23.16 ± 2.17^aA#^	33.47 ± 2.86^bB#^	31.33 ± 3.42^aAB#^	36.83 ± 5.78^bB#^	52.73 ± 4.52^bA#^	37.31 ± 1.70^bAC#^	33.27 ± 1.64^bBC#^	28.92 ± 2.87^bB#^

* *70 μmol m^–2^ s^–1^*;

** *130 μmol m^–2^ s^–1^*;

*** *220 μmol m^–2^ s^–1^*.

*Different letters indicate significant differences (p < 0.05) according to the Kruskal–Wallis test; small letters—differences between samples from three LI; capital letters—differences between samples from four culture durations measured in days, #,##—differences between samples derived from two temperature regimes within a single culture duration period*.

### Total Phenolic Content

**Figure 4:**
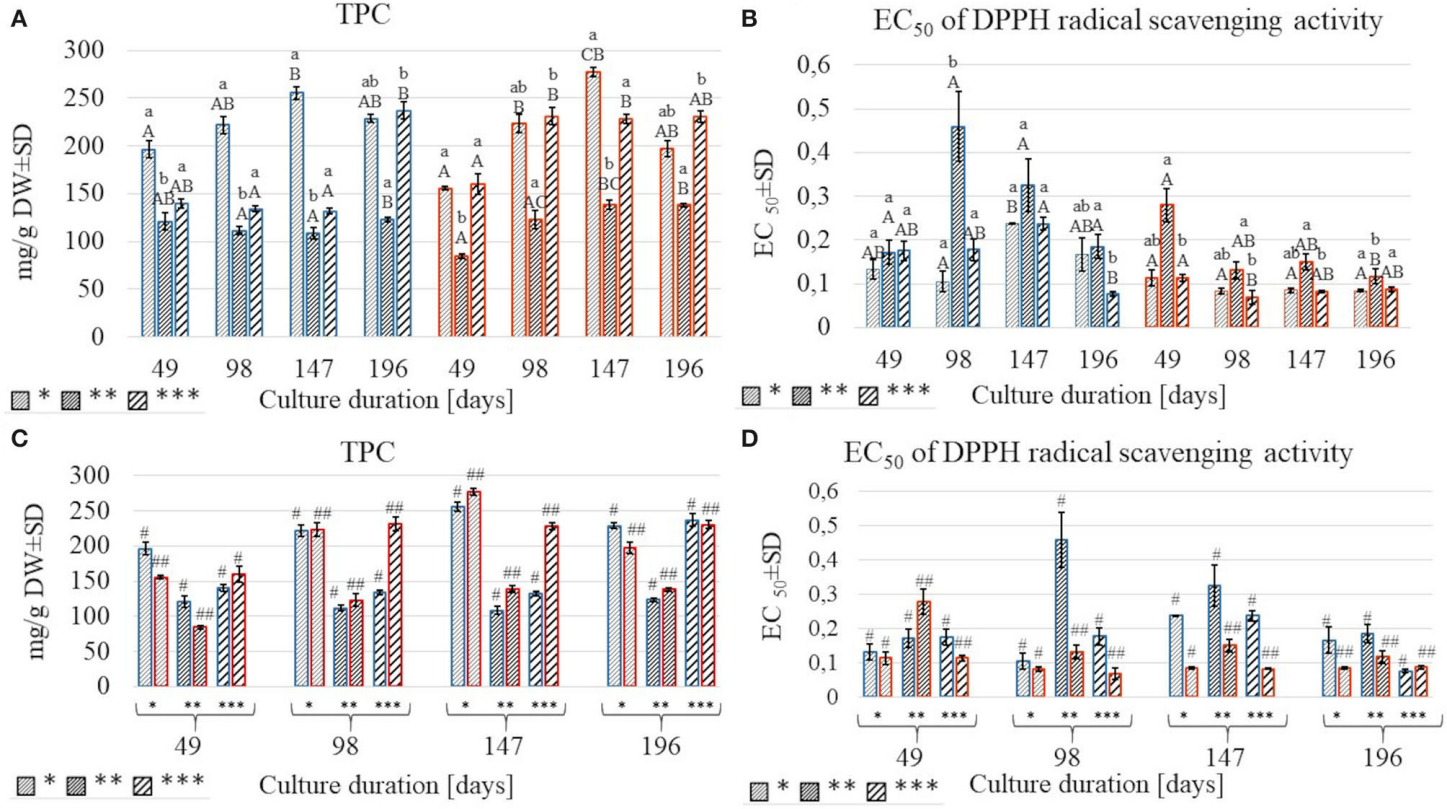
A comparison of total polyphenolic content (TPC) and EC_50_ values of DPPH test in *S.yangii* methanolic extracts from shoots cultured under three light intensities for four culture durations **(A,B)** and two temperature regimes **(C,D)**; LI: *70 μmol m^−2^ s^−1^; **130 μmol m^−2^ s^−1^; ***220 μmol m^−2^ s^−1^; blue: 25°C, red: 30°C. Statistical significance of differences was estimated using Kruskal–Wallis test at *p* < 0.05 and marked with different letters: small letters—differences between samples from three LI; capital letters—differences between samples from four culture durations measured in days; #, ##—differences between samples from two temperature regimes within a single culture duration period.

The effect of light on the TPC was non-linear. The lowest amounts (84.51–138.5 mg/g DW) were obtained under the moderate light intensity (130 μmol m^−2^ s^−1^), regardless of the impact of temperature and culture duration. At 25°C, the highest amounts of TPC were obtained under the lowest LI except for those detected in shoots harvested after 196 days, where TPC was comparable for 70 and 220 μmol m^−2^ s^−1^ (Figures 4A,C). At 30°C, TPC in shoots cultured under 220 μmol m^−2^ s^−1^ was like or higher than those obtained when 70 μmol m^−2^ s^−1^ was used, except for shoots harvested after 147 days of culture (Figure 4A). The effect of culture duration on TPC was also observed. The contents increased each time after the first three cultivation periods and decreased on the 196th day of harvest under all LI treatments at 30°C. The same effect was observed at the lowest LI conditions at 25 °C. The relative contribution analysis showed that TPC was more affected by the culture duration (11.848%) than by temperature (3.564%) (Figure 3, Supplementary Table 5).

### DPPH Radical Scavenging Activity

The results of anti-free radical activity (DPPH) showed similar trends as TPC, although some specific differences were observed. The highest EC_50_ value was obtained at moderate light, except for shoots harvested after 49 days at 25°C. Temperature significantly affected radical scavenging activity, with EC_50_ values being higher at 25°C compared to 30°C (Figures 4B,D). Culture duration also affected the DPPH results. That was confirmed by the relative contribution of variance (Figure 3, Supplementary Table 5).

### Non-volatile Metabolites

**Figure 5:**
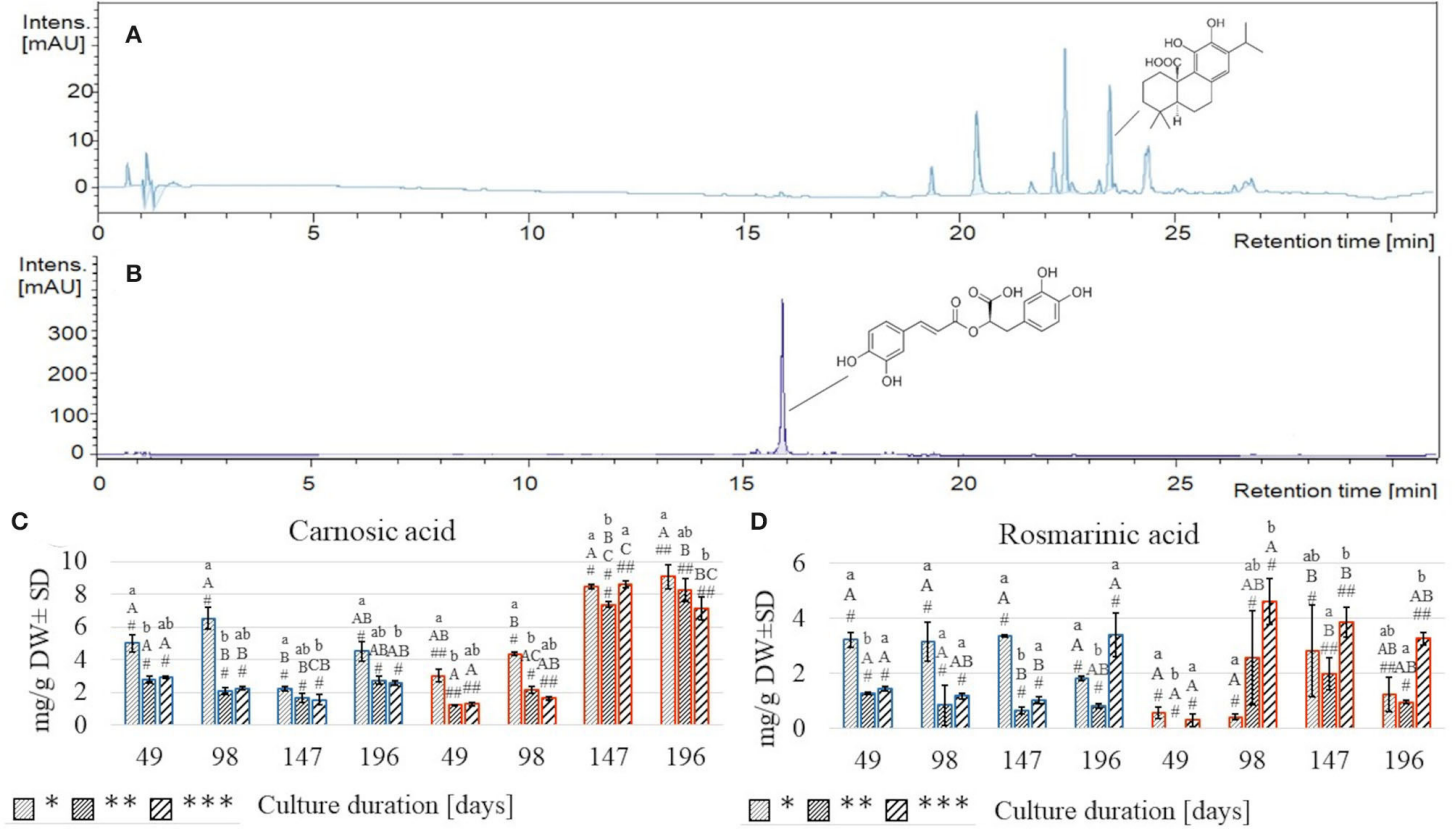
Chromatograms of *S. yangii* EtOAc extract acquired at 280 nm **(A)** and MeOH extract acquired at 320 nm **(B)**; the content of carnosic acid **(C)** and rosmarinic acid **(D)** in *S. yangii* shoots cultured under three LI and two temperature regimes. Significant differences were evaluated at *p* < 0.05 using the Kruskal–Wallis test and are marked with different letters: small letters—differences between samples from three LI; capital letters—differences between samples from four culture durations measured in days; #, ##—differences between samples from two temperature regimes within a single culture duration period; LI: *70 μmol m^−2^ s^−1^; **130 μmol m^−2^ s^−1^; ***220 μmol m^−2^ s^−1^; blue: 25°C, red: 30°C.

Extraction of plant material with two solvents of different polarities resulted in the separation of CA (Figure 5A), detected in the EtOAc extract, and RA (Figure 5B), revealed mostly in the MeOH extract.

Both compounds responded differently to the treatments (Figures 3, 5C,D, Supplementary Table 4). At 25°C, RA was more than twice higher at 70 μmol m^−2^ s^−1^ than at 130 and 220 μmol m^−2^ s^−1^ and remained relatively stable throughout the culture (3.15–3.35 mg/g DW) until the last harvest, at 196th day when it decreased rapidly (1.82 mg/g DW) (Figure 5D). Conversely, under high light intensity, RA content after gradual decrease after 49, 98, and 147 days (1.44; 1.19; 1.04 mg/g DW, respectively) increased more than thrice (3.39 mg/g DW) at the final harvest (day 196) (Figure 5D). These trends resembled the TPC results (Figures 4A,C). At 30°C, the response in RA content was diverse and different from the TPC, being the lowest of all samples on day 49 (0.001–0.56 mg/g DW), then on day 98 increased markedly under high light (nearly 15 times). On day 147, the RA content increased almost 7-times under low light, while under moderate and high light, it slightly decreased (Figure 5D).

CA content at 25°C was the highest under low light, reaching maximum value on day 98 (6.55 mg/g DW), dropped to ca. quarter of this value on day 147, and grew again to 4.52 mg/g DW on the last harvest (Figure 5C). Under moderate and high light regimes, CA content decreased from day 49 (2.80 and 2.93 mg/g DW) till day 147 (1.66 and 1.56 mg/g DW) and increased back to the initial content on day 196 (2.76 and 2.58 mg/g DW, respectively, for 130 and 220 μmol m^−2^ s^−1^) (Figure 5C).

At 30°C, the trend was completely different, showing a systematic increase in CA content with time, disregarding the illumination intensity. After two subsequent culture durations (49 and 98 days), the CA content was significantly higher (3.04, 4.37 mg/g DW, respectively) under the low LI compared to moderate and high LI (Figure 5C).

### Volatile Organic Compounds (VOCs)

Monoterpene hydrocarbons (α-thujene, α-pinene, camphene, β-pinene, α-phellandrene, β-myrcene, *p*-cymene, limonene, β-(*E*)-ocimene), oxygenated monoterpenes (1,8-cineole, camphor, borneol, terpinene-4-ol, α-terpineol) and acylated oxygenated monoterpenes (bornyl acetate, α-terpinyl acetate) as well as sesquiterpene hydrocarbons (α-gurjunene, β-caryophyllene, α-humulene, aromadendrene, alloaromadendrene, γ-muurolene), and oxygenated sesquiterpenes (globulol, τ-cadinol) were detected in hexane extracts of *S. yangii in vitro* shoots.

A total of 24 compounds constituted 65–95% of the VOCs contained in the extract (Supplementary Table 2). The most abundant compound of all VOCs was 1,8-cineol. Its relative content varied in the range of 13.9–22.9% at 25°C, and 10.1–18.7% at 30°C treatment (Table 3).

**Table 3:** The content of VOCs in hexane extracts (area % ± SD) of *S. yangii* shoots cultured *in vitro* in different LI and temperature regimes.

**RI lit**	**RT**	**Name**	49^*^	49^**^	49^***^	98^*^	98^**^	98^***^	147^*^	147^**^	147^***^	196^*^	196^**^	196^***^
**Temperature 25** **°** **C**														
937	6.31	α-Pinene	3.95 ± 0.01^aA#^	5.60 ± 1.49^bA#^	4.79 ± 1.05^bA#^	4.12 ± 0.56^aA#^	5.19 ± 1.63^bA#^	5.92 ± 0.74^bA#^	3.23 ± 0.15^aA#^	6.85 ± 0.23^bA#^	6.53 ± 0.85^bA#^	4.00 ± 0.76^aA#^	10.00 ± 0.47^bA#^	5.37 ± 0.31^aA#^
1032	9.05	Limonene	10.18 ± 0.68^aA#^	9.06 ± 1.21^aA#^	8.84 ± 1.42^aAB#^	7.72 ± 0.75^aA#^	7.15 ± 1.46^aA#^	9.89 ± 0.60^aAB#^	3.52 ± 0.20^aA#^	7.49 ± 0.86^aA#^	7.09 ± 1.17^aB#^	9.61 ± 1.58^aA#^	11.89 ± 0.58^abA#^	13.78 ± 0.29^bA#^
1036	9.13	1,8-Cineol	17.02 ± 0.13^aA#^	15.71 ± 1.98^aAB#^	18.60 ± 1.15^aA#^	17.73 ± 1.45^aA#^	13.96 ± 1.69^aA#^	18.40 ± 1.64^aA#^	16.63 ± 0.93^aA#^	16.68 ± 0.34^aAB#^	19.21 ± 2.84^aA#^	18.33 ± 2.37^aA#^	18.47 ± 1.43^aB#^	22.89 ± 1.75^aA#^
1146	12.87	Camphor	6.78 ± 0.28^aAB#^	3.97 ± 0.08^aA#^	3.82 ± 0.21^aA#^	4.50 ± 0.98^aA#^	3.44 ± 0.37^aAB#^	3.07 ± 0.38^aA#^	6.94 ± 0.10^aB#^	2.98 ± 0.11^aAB#^	3.21 ± 0.30^aA#^	6.27 ± 0.28^aAB#^	2.86 ± 0.12^bB#^	4.23 ± 0.22^abA#^
1419	21.71	β-Caryophyllene	4.64 ± 0.50^abA#^	3.65 ± 0.42^aA#^	5.72 ± 0.22^bAB#^	5.09 ± 0.40^aA#^	4.28 ± 0.50^aA#^	5.01 ± 0.28^aAB#^	4.94 ± 0.14^aA#^	3.86 ± 0.08^bA#^	4.62 ± 0.08^abB#^	6.59 ± 0.52^aA#^	3.23 ± 0.03^bA#^	6.07 ± 0.32^abA#^
1454	22.48	α-Humulene	6.12 ± 0.60^aA#^	4.15 ± 0.52^aA#^	6.32 ± 0.19^aAB#^	7.14 ± 0.81^aA#^	4.56 ± 0.41^bA#^	5.46 ± 0.37^abAB#^	6.57 ± 0.19^aA#^	4.25 ± 0.17^bA#^	4.89 ± 0.12^abB#^	9.26 ± 0.86^aA#^	3.63 ± 0.14^bA#^	7.28 ± 0.04^abA#^
1461	22.64	Alloaromadendrene	6.86 ± 0.79^aAB#^	11.16 ± 1.22^bA#^	8.74 ± 0.34^abA#^	8.59 ± 0.76^abA#^	11.32 ± 1.32^aA#^	6.79 ± 0.51^bAB#^	0.24 ± 0.03^aB#^	9.41 ± 0.45^bA#^	6.43 ± 0.19^abAB#^	0.34 ± 0.14^aAB#^	9.23 ± 0.35^aA#^	0.32 ± 0.02^aB#^
1642	25.67	τ-Cadinol	1.83 ± 0.25^aA#^	nd	1.09 ± 0.14^aA#^	2.05 ± 0.42^aAB#^	nd	1.09 ± 0.06^aA#^	3.67 ± 0.21^aB#^	nd	1.12 ± 0.06^aA#^	3.22 ± 0.40^aAB#^	nd	2.98 ± 0.02^aA#^
**Temperature 30** **°** **C**														
937	6.31	α-Pinene	7.03 ± 1.18^aA#^	10.23 ± 0.69^bA#^	7.90 ± 0.48^bA#^	4.79 ± 1.82^aA##^	9.65 ± 1.68^bA#^	5.72 ± 1.09^aAB##^	3.54 ± 0.11^aA#^	7.11 ± 0.90^bA##^	5.63 ± 0.15^aB##^	5.07 ± 0.23^aA#^	6.61 ± 0.20^aA#^	6.29 ± 0.03^aAB#^
1032	9.05	Limonene	10.50 ± 1.54^abA#^	8.25 ± 0.62^aA#^	11.73 ± 0.86^bAB##^	11.81 ± 2.16^aA#^	10.55 ± 0.17^aA#^	9.73 ± 1.16^aA#^	6.81 ± 0.22^aA##^	7.63 ± 1.09^abA#^	11.67 ± 0.41^bAB##^	11.77 ± 0.41^abA##^	8.25 ± 0.13^aA##^	14.63 ± 0.21^bB^^##^
1036	9.13	1,8-Cineol	13.54 ± 0.83^abA#^	10.16 ± 0.45^aA##^	15.23 ± 0.43^bA#^	16.20 ± 0.73^aAB#^	11.34 ± 0.93^aA##^	17.90 ± 0.61^aAB#^	18.74 ± 0.04^aB##^	11.91 ± 0.74^bA##^	18.12 ± 0.49^abB#^	16.39 ± 0.18^abAB#^	12.19 ± 0.19^aA##^	17.22 ± 0.21^bAB##^
1146	12.87	Camphor	7.61 ± 0.27^aA##^	5.55 ± 0.06^abA##^	5.27 ± 0.23^bA##^	9.85 ± 0.71^aA#^	7.63 ± 0.09^aB##^	8.02 ± 0.45^aB##^	9.88 ± 0.08^aA##^	7.13 ± 0.18^abAB##^	6.09 ± 0.17^bAB##^	8.17 ± 0.14^aA#^	5.86 ± 0.17^aAB##^	5.73 ± 0.09^aAB##^
1419	21.71	β-Caryophyllene	7.45 ± 0.65^abA##^	3.83 ± 0.25^aA#^	8.46 ± 0.50^bA##^	7.73 ± 1.02^aA#^	3.69 ± 0.43^aA#^	7.47 ± 0.64^aA##^	7.90 ± 0.11^abA##^	4.73 ± 0.69^aA##^	8.46 ± 0.25^bA##^	8.30 ± 0.13^abA##^	5.00 ± 0.06^aA##^	8.58 ± 0.15^bA##^
1454	22.48	α-Humulene	8.84 ± 0.90^aA##^	4.19 ± 0.31^aA#^	8.73 ± 0.56^aA##^	8.38 ± 1.14^aA#^	3.89 ± 0.49^aA#^	7.47 ± 0.55^aA##^	8.58 ± 0.16^aA##^	4.68 ± 0.51^bA#^	8.19 ± 0.31^abA##^	8.60 ± 0.04^aA#^	4.66 ± 0.08^bA##^	8.08 ± 0.17^abA#^
1461	22.64	Alloaromadendrene	0.33 ± 0.04^abAB##^	11.08 ± 0.67^aA#^	0.25 ± 0.02^bA##^	0.22 ± 0.10^aA##^	10.37 ± 1.37^aA#^	0.28 ± 0.00^aA##^	0.44 ± 0.02^abB##^	11.86 ± 1.03^aA##^	0.43 ± 0.01^bA#^	0.36 ± 0.05^abAB#^	11.53 ± 0.10^aA#^	0.30 ± 0.03^bA#^
1642	25.67	τ-Cadinol	5.25 ± 0.77^aA##^	0.14 ± 0.00^bA#^	5.62 ± 0.42^aAB##^	5.40 ± 0.67^aA#^	nd	6.79 ± 0.42^aA##^	6.52 ± 0.09^aA##^	0.18 ± 0.01^bA##^	6.36 ± 0.22^aAB##^	4.74 ± 0.09^aA##^	nd	4.98 ± 0.12^aB##^

* *70 μmol m^–2^ s^–1^*;

** *130 μmol m^–2^ s^–1^*;

*** *220 μmol m^–2^ s^–1^ for 49, 98, 147, and 196—culture duration [days]; RI, retention index, RT, retention time. n = 3*.

*Significant differences were evaluated at p < 0.05 using Kruskal–Wallis test and are marked with different letters: small letters—differences between samples from three LI; capital letters—differences between samples from four culture durations measured in days*;

*#, ##—differences between samples from two temperature regimes within a single culture duration period*.

The other major compounds in all samples were limonene, β-caryophyllene, α-humulene, α-pinene, camphor, borneol, bornyl acetate, and terpinyl acetate (Supplementary Table 2).

All experimental treatments influenced the VOCs composition in terms of their quality and quantity, whereas LI was the most significant (Table 3, Figure 3; for detailed data see Supplementary Tables 2, 4). LI of 130 μmol m^−2^ s^−1^ resulted in the highest content of alloaromadendrene, α-pinene, borneol, and bornyl acetate, compared to lower and higher LI at both temperatures (Table 3). The differences in the alloaromadendrene content were significantly larger at the higher temperature (Table 3).

The VOCs analysis regarding groups of compounds showed oxygenated monoterpenes and sesquiterpenes hydrocarbons as predominant at 25°C in all light regimes. At higher temperature after 49 days, monoterpenes hydrocarbons were similar (70 μmol m^−2^ s^−1^) or more abundant (130 and 220 μmol m^−2^ s^−1^) than oxygenated. At the second harvest (98 days), monoterpene hydrocarbons were predominant only under the moderate LI, and at 147 days became lower than oxygenated monoterpenes disregarding illumination intensity. At 30°C, also the sesquiterpenes differed in a light-dependent manner with oxygenated being the highest under 70 and 220 μmol m^−2^ s^−1^ and hydrocarbons under 130 μmol m^−2^ s^−1^ in each harvest (Supplementary Table 2).

### Statistical Analysis

To follow the impact of LI, temperature, and culture duration on plant metabolic and morphogenic response, the relative contribution of variance was performed. Each factor as well as their interactions were investigated (Figure 3). The analysis showed that the contents of selected VOCs were more affected by light than by any other factor. In the case of non-volatile compounds—a combination of two factors was more evident. It was light and temperature combined effect and light as a single factor for RA while for CA mainly the culture duration was followed by light intensity and temperature as single factors (Supplementary Table 4). Additionally, light as a single factor significantly influenced TPC content. Moreover, chlorophylls and carotenoids content as well as the chl a + chl b / carotenoids ratio were mostly affected by the LI. On the other hand, the chlorophyll a to b ratio (chl a/chl b) and DPPH results, were more affected by temperature. TBARS were almost equally affected by a combination of temperature and culture duration as well as light, as a single factor (Supplementary Table 5). The morphogenetic response was mainly affected by light, especially in the case of the length of axillary shoots, mean number of nodes per 1 cm of axillary shoot, and mean mass of shoot. In turn, the mean number of axillary shoots per explant was mostly affected by the temperature, while the roots length by culture duration (for detailed data see Supplementary Table 3).

**Figure 6:**
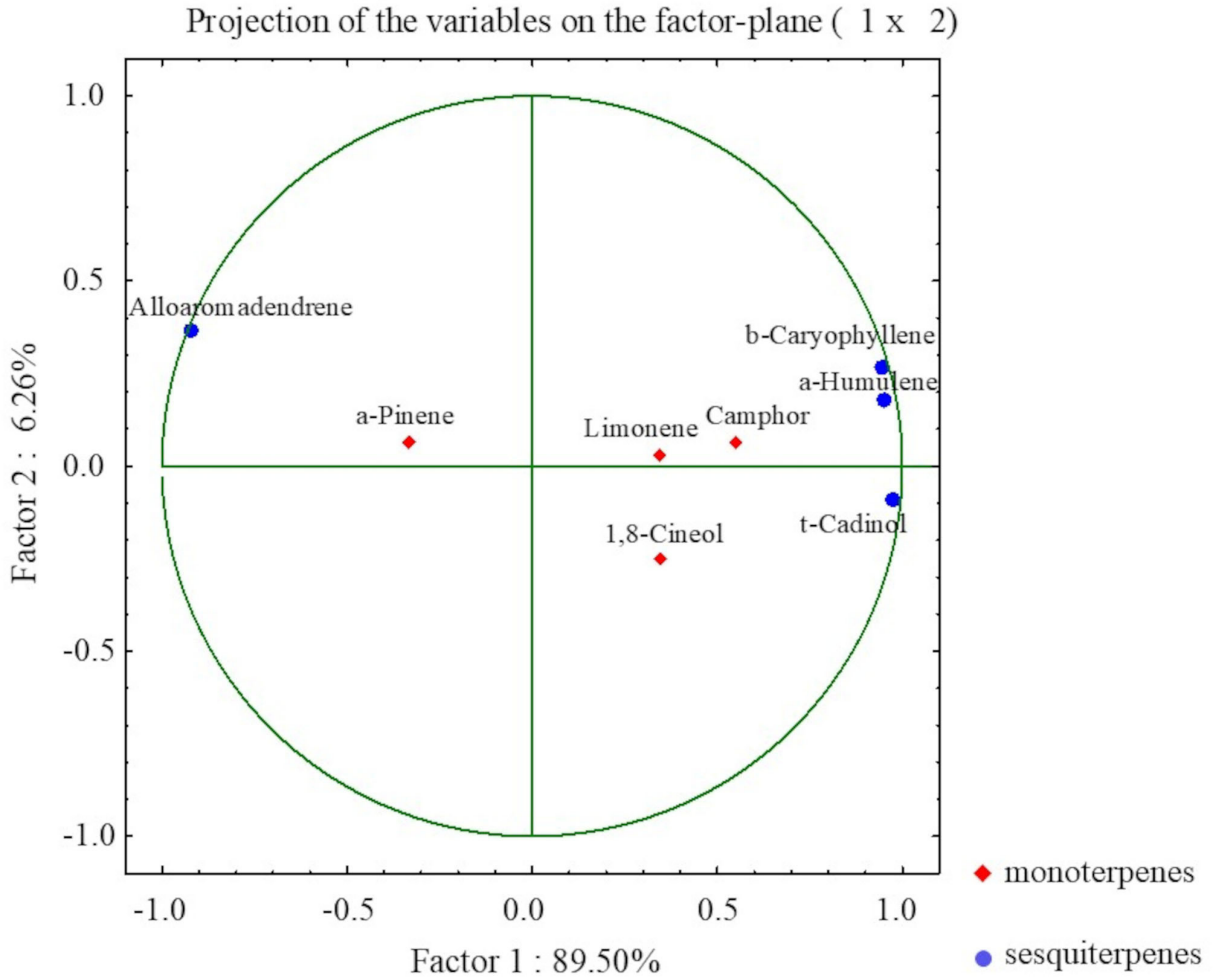
The PCA presenting relationships between selected VOCs in *S. yangii in vitro* shoots in response to light intensity, temperature, and culture duration.

Furthermore, to determine the correlation between the most abundant volatile organic compounds, a PCA was performed. The PCA indicated 4 components explaining 100% of the total variance, with the first two PCs explaining 89.5 and 6.26%, respectively (Figure 6). For each factor, a principal component loading of more than 1.37 was considered significant.

The PCA distinguished two main groups of VOCs within sesquiterpenoid and monoterpenoid constituents that were negatively correlated to each other. Within sesquiterpenoids, a strong negative correlation was observed between alloaromadendrene and the group composed of β-caryophyllene, α-humulene, and τ-cadinol. Within monoterpenoids, a negative correlation occurred between α-pinene and the group formed by limonene, camphor, and 1,8-cineol. Moreover, α-pinene was positively correlated to alloaromadendrene, whereas limonene, camphor, and 1,8-cineol to τ-cadinol, α-humulene, and β-caryophyllene (Figure 6).

## Discussion

*S. yangii* was selected as a suitable object for the light- and heat-stimulation *in vitro* experiments due to its origin and metabolic profile. The phytochemical analysis of both volatile and non-volatile compounds revealed large differences between samples after three different LI and two different temperature treatments. The experiments were carried out for a relatively long time (the last harvest at 196 days). Over these 7 months, the morphological and metabolic response of the tested plants was developed. The most rapid changes were observed in the content of specialized metabolites.

As both investigated factors, the intensities of light and the temperatures were of moderate magnitude, the morphology of the plants was not strongly disturbed. Only the typical lengthening of the internodes in lower light conditions and their shortening with increasing light intensity were observed (Fiorucci and Frankhauser, 2017). Similarly, in the case of the leaf lamina, the area was larger when higher light intensity was used, but lighter green and containing chlorophyll-free sectors at the same time (Bugbee, 2016).

The photosynthetic pigments content suggested an overlapping effect of factors such as light exposure, temperature, and culture duration on the tested parameters. Plant photosynthetic pigments are directly involved in light capturing, being also the most vulnerable to stress-induced ROS. Alterations in chlorophylls content indicate functional pigment adaptations of the photosynthetic apparatus to certain light conditions. These changes are associated with the functional rearrangement of photosynthesis centers. Chlorophyll b occurs exclusively in light harvesting complex (LHC) antennas whereas chlorophyll a is present also in reaction centers of the photosystems (PSI and PSII) (Lichtenthaler and Buschmann, 2001; Tokarz et al., 2018). The chlorophyll a/b ratio is an indicator of functional pigment equipment, and the change of this value converts to antenna size. A decrease in total chlorophyll content observed in *S. yangii* in high light indicates severe pigment destruction, while changes in chlorophyll a/b ratio suggest interference in the antenna size. Light-induced chlorophyll breakdown was previously observed in numerous studies concerning light intensity impact on plant growth and development (Alvarenga et al., 2015; Lazzarini et al., 2018; de Hsie et al., 2019). Light-induced remodeling of LHC also affects carotenoids, which are part of antenna subunits. Carotenoids play a crucial role in non-photochemical quenching by converting excess photon energy into heat, instead of transferring it to an already excited PSII center. An increase in carotenoid content is associated with compensation of energy in the xanthophyll cycle, however, its decrease may be connected with the reduction of photosynthetic apparatus linked with LHC. In addition to the content, the ratio of chlorophylls to carotenoids indicates damage to the photosynthetic apparatus caused by stress or senescence (Lichtenthaler and Buschmann, 2001). Our results showed that this ratio in *S. yangii* shoot cultures was lower in higher LI (220 μmol m^−2^ s^−1^), which may be related to the stepped-up senescence and intense remodeling of the photosynthetic apparatus.

To monitor the redox conditions, we estimated TBARS. The accumulation of TBARS indicates the formation of lipid peroxidation by-products due to cell membrane damage. In our study, a combination of temperature and culture duration as well as light as a single factor significantly influenced TBARS accumulation. Co-stress caused by light- and heat-induced lipid peroxidation was previously described, but in more extreme conditions maintained for a shorter time (Gerganova et al., 2016; Chen et al., 2017; Zhou et al., 2020). TBARS elevation is detectable after 3 h of acute heat and high light coincidence (Chen et al., 2017) and gains with stress factor duration (Rysiak et al., 2021).

To assess the metabolic response of *S. yangii* microshoots under the tested conditions, selected non-volatile and volatile compounds were quantified using chromatographic techniques.

*S. yangii* is rich in specialized metabolites from different chemical classes. Among the non-volatile compounds, CA (abietane-like oxygenated diterpene), also called salvin, is one of the most abundantly produced (Bielecka et al., 2021). In our experiments, CA content was higher when plants were cultured at a higher temperature (30°C). Moreover, much higher levels of this compound were recorded after the longer cultivation periods, i.e., 147 and 196 days. Considering the obtained results, we postulate a role of this compound in complex protective reactions of *S. yangii* shoots, in particular against cell damage caused by lipid degradation. The antioxidant activity of CA was previously recognized for other Lamiaceae species, especially plants from harsh habitats with high sun exposure and large temperature fluctuations (Birtić et al., 2015). Moreover, after the natural degradation of this compound, further metabolites such as carnosol, rosmanol, and isorosmanol still display strong antioxidant activity. The sustained activity is associated with the presence of two hydroxyl groups in C-11 and C-12 positions in the derivatives (Tounekti and Munné-Bosch, 2012). CA was found in aerial parts, especially leaves, and was more abundant in young plants (del Baño et al., 2003). At the cellular level, it is located mainly in chloroplast membranes, although about 10% of its total content is found in the trichomes. According to previous data, CA content can be influenced by such factors as growing conditions including low water availability, UV-B radiation, or foliar spray with kinetin (10 μM) (Tounekti and Munné-Bosch, 2012). The content of CA was also correlated to the photoperiod and seasonal changes, although the data collected were ambiguous. The studies by Hidalgo et al. (1998) on *Rosmarinus officinalis* showed the largest content of CA in July, while by Luis and Johnson (2005), the lowest content of CA was recorded during summer. In Mediterranean conditions of high radiation and water stress, the CA content was lower, while its oxidative metabolites were detected in higher proportions. These results suggested a stress-induced CA depletion (Tounekti and Munné-Bosch, 2012). CA and its metabolites deserve special attention due to their rare occurrence in the plant kingdom. CA was found in only 9 out of 220 Lamiaceae genera (Birtić et al., 2015). Due to its proven antioxidant effect, CA is increasingly used as a food additive. Both CA and carnosol are used under the code E392 as active antioxidants in foods in Europe (Birtić et al., 2015).

RA is the most abundant non-volatile polyphenolic compound present in the aerial parts. It was produced in relatively low amounts (0.31–4.61 mg/g d.w.), compared to other Lamiaceae. LI as well as the combination of LI and temperature shared the highest relative contribution to RA content in our studies. The highest quantities of RA and TPC were detected under the lowest and the highest LI (70 and 220 μmol m^−2^ s^−1^, respectively). Previously reported data on the *Orthosiphon stamineus* showed increased TPC accumulation under low LI treatment and was linked to increased availability of a biosynthesis precursor—phenylalanine (Ibrahim and Jaafar, 2012). The relatively low content of TPC under moderate LI was observed also by other researchers, who explained it by the higher biomass production and structural rearrangement of metabolites, such as carbohydrates, that are intensively produced during plant development (del Baño et al., 2003).

Polyphenolic compounds present the well-known antioxidant activity that is more related to their composition than to their content (Yeddes et al., 2019). In our studies, the results of the DPPH test showed the highest radical scavenging activity for the extracts of shoots cultured under moderate LI. Whereas, the content of TPC and RA in these samples was relatively low. It may be assumed that the strength of the activity was supported by other compounds present in the tested extracts, such as CA. The latter was found to be a much stronger radical scavenger compared to RA (Birtić et al., 2015).

The second important group of compounds in *S. yangii* is VOCs. They are mainly terpenoids that make up the essential oil contained in the glandular trichomes formed on the aerial organs. VOCs consist of several dozen substances from four main chemical groups, i.e., monoterpene hydrocarbons, oxygenated monoterpenes, sesquiterpene hydrocarbons, and oxygenated sesquiterpenes. In the VOCs mixture, a slight predominance of 1,8-cineole, limonene, Δ3-carene, or β-caryophyllene was reported (Jassbi et al., 1999; Erdemgil et al., 2007).

In our study, 1,8-cineole, also known as eucalyptol, was produced in the highest amounts. Its content ranged between 14 and 23% and 10 and 18.7% at 25 and 30°C, respectively. Along with camphor and borneol, 1,8-cineole represented the most abundant group of oxygenated monoterpenes predominant at 25°C. At higher temperatures, after the first harvest, the ratio between oxygenated and monoterpenes hydrocarbons reversed, favoring the more reduced compounds. The fluctuations in the structural rearrangements of monoterpenes indicate possible adaptive reactions triggered by mild thermal stress. For instance, monoterpenes hydrocarbons often take part in plant defense reactions against oxidative stress, including thermal stresses (Fantaye, 2014). Stress conditions elevate the reducing power of plant metabolic reactions resulting in higher proportions of reduced compounds. The overproduction of NADPH was previously reported in the case of environmental stress supporting our observations (Corpas and Barroso, 2014).

Our studies showed that sesquiterpene hydrocarbons were more abundant than oxygenated in both temperature regimes. Moreover, two groups within sesquiterpenoids were separated in terms of LI treatment. The observed changes did not correspond to the reaction observed in the case of monoterpenes. It may be correlated to the cell compartment production sites—monoterpenes are biosynthesized in chloroplasts, while sesquiterpenes are produced in the cytosol. Alloaromadendrene content was influenced by LI more than by any other factor. This compound was produced in significantly greater amounts in shoots grown under moderate LI (130 μmol m^−2^ s^−1^), than under the lowest and the highest LI, where its content was at least several times lower. Such a relationship was observed for samples from cultures at both temperature treatments. In turn, three other sesquiterpenes, namely β-caryophyllene, α-humulene, and τ-cadinol showed the reverse trend to alloaromadendrene in terms of the LI effect.

The PCA analysis revealed the association between two groups of compounds: the first composed of α-humulene, β-caryophyllene, τ-cadinol and the second consisted of alloaromadendrene. The first group was represented by the humulene and cadalane derivatives, while the second was composed of structures of dimethyl cyclopropane ring fused to hydroazulene skeleton. As both are farnesyl diphosphate derivatives, a different cyclization scheme is involved. The C10-1 cyclization forms germacryl cation, a precursor of aromadendrene derivatives, while C11-1 cyclization is involved in humulene cation formation (Vattekkatte et al., 2018). Interestingly, the shift between cadalane- and germacrene-based products was previously reported in studies on sesquiterpene synthase isolated from *Medicago truncatula* (Garms et al., 2010). In that case, the pH value determined product specificity showing germacrene domination in high pH, while cadalane in a preference for low pH values. Moreover, alterations in divalent ions concentration may also interfere with the catalytic activity of terpene synthases affecting cyclization patterns. Local pH variations occurring as a result of stress-induced redox imbalance may interrupt plant biosynthesis in a kind of adverse way of regulation (Ruan et al., 2016). Fluctuation in the VOCs content due to the various environmental conditions was observed in many plant species, although it is still a poorly understood phenomenon. Our statistical analysis revealed correlations of certain compounds between each other but more prominently between compounds from different groups. A positive relation between TBARS and some monoterpenes like camphene, α-pinene, p-cymene, and α-phellandrene suggested their role in stress reaction. As it was previously reported, stress may induce a high reducing power that affects the metabolic processes, favoring the synthesis of highly reduced compounds (Corpas and Barroso, 2014). Also, monoterpenes may be involved in the membrane stabilization by direct action against ROS or by interactions on lipid-lipid, lipid-protein, and protein-protein levels (Lazzarini et al., 2018). On the other hand, the metabolic cost of monoterpene production is higher than that of diterpenes, which may affect the content of CA (Gershenzon, 1994). It can be speculated that plants under environmental stress conditions produce larger amounts of compounds that are easier to synthesize. In *Salvia* sp., it would be CA rather than the monoterpene constituents of the essential oil.

## Conclusions

The structural diversity within various classes of natural products with a broad spectrum of bioactivities, undoubtedly helps plants, such as *S. yangii*, to live in harsh environmental conditions. The causes of the accumulation of individual compounds are difficult to trace, although stimulation with selected environmental factors allows to observe some trends. The results of our experiments pointed to CA, which can be considered as one of the marker compounds that appears in a response to long-lasting stimulation of *S. yangii* shoots with increased temperature and intense light. Also, the altered composition of essential oil, especially of a few sesquiterpenes (alloaromadendrene, β-caryophyllene, α-humulene, and τ-cadinol) could suggest adaptive reactions of this plant species to given culture conditions. Hence, the results of this study provided new information on the metabolic profile plasticity in *S. yangii* and enriched the existing knowledge on other plants species from severe habitats.

## Data Availability Statement

The raw data supporting the conclusions of this article will be made available by the authors, without undue reservation.

## Author Contributions

WK, AM, and SZ contributed to conception and design of the study, wrote the first draft of the manuscript, and co-worked to complete the final version. WK performed the experiments and measurements, collected, processed and curated the data, interpreted the results, performed the statistical analysis, prepared the draft of the manuscript parts, and prepared the results presentation-tables and graphics. SZ supervised and participated in the experimental work. AM and SZ participated in the interpretation of experimental results and statistical analysis and advised on the results presentation. All authors contributed to manuscript revision, read, and approved the submitted version.

## Funding

This study was funded by the Wroclaw Medical University grant for young researchers Influence of illumination spectra on metabolic profile in *Salvia yangii* BT Drew number SUBK.D030.22.008 to WK. The Open Access publication was additionally supported by Wroclaw Medical University subvention Grant No. SUBZ.D030.22.017 and the Wroclaw Medical University Scientific Board of Pharmaceutical Sciences.

## Conflict of Interest

The authors declare that the research was conducted in the absence of any commercial or financial relationships that could be construed as a potential conflict of interest.

## Publisher's Note

All claims expressed in this article are solely those of the authors and do not necessarily represent those of their affiliated organizations, or those of the publisher, the editors and the reviewers. Any product that may be evaluated in this article, or claim that may be made by its manufacturer, is not guaranteed or endorsed by the publisher.

## Abbreviations

CA: carnosic acid
LI: light intensity
LHC: light harvesting complex
PS: photosystem
RA: rosmarinic acid
ROS: reactive oxygen species
VOCs: volatile organic compounds.
