# Supplementary material for: Light Intensity and Temperature Effect on Salvia yangii (B. T. Drew) Metabolic Profile in vitro
Source: Front Plant Sci. 2022 May 13;13:888509. doi: 10.3389/fpls.2022.888509 (PMC9136318; doi:10.3389/fpls.2022.888509)
Supplement: Supplementary file 1 [file Data_Sheet_1.PDF]

Supplementary tables

Light intensity and temperature effect on *Salvia yangii* (B.T.Drew) metabolic profile *in vitro*

Weronika Kozłowska, Adam Matkowski, Sylwia Zielińska

Table 1S. The content of chlorophyll a, b and carotenoids as well as chl a/chl b ratio and total chlorophyll to total carotanoids ratio (Chl a+Chl b)/ Car in *S. yangii* shoots cultured in different light intensities (LI), temperature (T) and culture duration (CD) in days; *70 µmol m^{-2} s^{-1}; **130 µmol m^{-2} s^{-1}; ***220 µmol m^{-2} s^{-1}. Different letters indicate significant differences ($p<0.05$) according to the Kruskal-Wallis test; small letters - differences between samples derived from three LI; capital letters – differences between samples derived from four culture durations measured in days. #, ## - differences between samples derived from two temperature regimes within a single culture duration period.

T	CD	LI	**Chl a**	**Chl b**	**Car**	**Chl a/Chl b**	**(Chl a+Chl b)/ Car**
**25°C**	**49**	*	6.82±0.67$^{aAB\#}$	2.31±0.26$^{bAB\#}$	1.66±0.17$^{aAB\#}$	3.37±0.12$^{aAC\#}$	5.50±0.07$^{aAB\#}$
		**	6.56±0.53$^{aA\#}$	2.04±0.19$^{abA\#\#}$	1.62±0.12$^{aA\#}$	3.62±0.04$^{bA\#}$	5.30±0.07$^{bA\#}$
		***	6.34±0.27$^{aA\#}$	1.96±0.10$^{aA\#}$	1.58±0.09$^{aA\#}$	3.59±0.10$^{bA\#}$	5.24±0.09$^{bA\#}$
	**98**	*	7.01±0.41$^{aB\#}$	2.21±0.16$^{aB\#}$	1.65±0.14$^{aA\#}$	3.63±0.09$^{aAB\#}$	5.60±0.16$^{abB\#}$
		**	6.43±0.14$^{aAB\#}$	2.16±0.06$^{aAB\#}$	1.61±0.04$^{aA\#}$	3.46±0.10$^{bA\#}$	5.35±0.13$^{aB\#}$
		***	5.10±0.44$^{bAB\#}$	1.66±0.24$^{bAB\#}$	1.43±0.27$^{aA\#}$	3.39±0.21$^{aAB\#}$	4.81±0.43$^{bAB\#}$
	**147**	*	7.67±0.60$^{aA\#\#}$	2.74±0.37$^{aA\#\#}$	1.85±0.12$^{aB\#\#}$	3.23±0.11$^{aC\#}$	5.61±0.18$^{aA\#}$
		**	7.21±0.27$^{aB\#}$	2.27±0.08$^{aAB\#}$	1.76±0.07$^{aA\#}$	3.76±0.06$^{bA\#}$	5.38±0.03$^{aA\#}$
		***	2.61±0.55$^{bC\#\#}$	0.87±0.18$^{bC\#\#}$	0.78±0.15$^{bB\#\#}$	3.49±0.09$^{cAB\#}$	4.43±0.10$^{bB\#}$
	**196**	*	7.50±0.05$^{abBC\#}$	2.50±0.03$^{abBC\#}$	1.77±0.02$^{abA\#}$	3.82±0.04$^{aB\#}$	5.86±0.06$^{aA\#}$
		**	7.16±0.84$^{aAB\#}$	2.49±0.33$^{aB\#}$	1.71±0.22$^{aA\#\#}$	3.28±0.10$^{bB\#}$	5.66±0.04$^{aB\#}$
		***	3.47±0.11$^{bBC\#}$	1.17±0.05$^{bBC\#}$	0.90±0.04$^{bB\#}$	3.44±0.09$^{abB\#}$	5.18±0.16$^{bA\#}$
**30°C**	**49**	*	7.81±0.23$^{aA\#}$	3.04±0.11$^{aA\#}$	1.86±0.08$^{aA\#}$	3.02±0.05$^{aA\#\#}$	5.86±.024$^{aA\#\#}$
		**	5.14±0.34$^{bA\#}$	2.10±0.15$^{bA\#\#}$	1.31±0.07$^{bAB\#}$	2.87±0.03$^{bA\#\#}$	5.53±0.08$^{bA\#\#}$
		***	3.77±0.85$^{cA\#}$	1.43±0.31$^{cA\#}$	1.08±0.18$^{bA\#}$	3.03±0.04$^{aA\#\#}$	4.79±.029$^{cA\#\#}$
	**98**	*	7.73±0.33$^{aB\#}$	2.92±0.11$^{aB\#}$	1.82±0.09$^{aB\#}$	3.13±0.09$^{aB\#\#}$	5.86±0.09$^{aAB\#\#}$
		**	6.72±0.26$^{bB\#}$	2.61±0.12$^{bB\#}$	1.67±0.07$^{bB\#}$	2.93±0.04$^{bB\#\#}$	5.59±0.15$^{bB\#\#}$
		***	2.92±0.11$^{cB\#}$	1.11±0.05$^{cB\#}$	0.86±0.04$^{aB\#}$	3.12±0.06$^{bAB\#\#}$	4.71±0.09$^{cB\#\#}$
	**147**	*	7.50±0.23$^{aAB\#\#}$	2.60±0.10$^{aB\#\#}$	1.80±0.07$^{aB\#\#}$	2.88±0.10$^{aAB\#\#}$	5.61±0.11$^{aAB\#}$
		**	5.96±0.12$^{bA\#}$	2.05±0.04$^{bA\#}$	1.48±0.03$^{bA\#}$	3.43±0.12$^{abB\#\#}$	5.40±0.03$^{abAC\#}$
		***	3.33±0.56$^{cA\#\#}$	1.09±0.24$^{cA\#\#}$	0.88±0.09$^{cA\#\#}$	3.60±0.27$^{bB\#}$	4.98±0.42$^{bA\#\#}$
	**196**	*	6.94±0.55$^{aB\#}$	2.32±0.18$^{aB\#}$	1.68±0.09$^{aB\#}$	3.37±0.06$^{aAC\#\#}$	5.49±0.13$^{aB\#\#}$
		**	5.65±0.44$^{bA\#}$	2.19±0.15$^{aA\#}$	1.53±0.11$^{abA\#\#}$	3.00±0.07$^{bABC\#\#}$	5.12±0.03$^{bBC\#\#}$
		***	4.88±0.61$^{bAB\#}$	1.70±0.25$^{bAB\#}$	1.31±0.13$^{bAB\#}$	3.32±0.09$^{aB\#\#}$	4.99±0.17$^{bA\#}$

Table 2S. The content of VOCs in hexane extracts (area % ± SD) of *S. yangii* shoots cultured *in vitro* in different LI and temperature regimes; *70 µmol m^{-2} s^{-1}, **130 µmol m^{-2} s^{-1}, ***220 µmol m^{-2} s^{-1}; 49, 98, 147, and 196 – culture duration [days]; RI – retention index, RT – retention time. n=3. Different letters indicate significant differences (p<0.05) according to the Kruskal-Wallis test; small letters - differences between samples derived from three LI; capital letters – differences between samples derived from four culture durations measured in days. #, ## - differences between samples derived from two temperature regimes within a single culture duration period.

Temperature 25°C															
**RI lit**	**RI calc**	**RT**	**Name**	**49***	**49****	**49*****	**98***	**98****	**98*****	**147***	**147****	**147*****	**196***	**196****	**196*****
930	937	6.13	**α-Thujene**	0.31±0.01<br>aA#	0.41±0.03<br>aAB#	0.43±0.07<br>aA#	0.29±0.04<br>aA#	0.46±0.05<br>bA#	0.38±0.05<br>abB#	0.27±0.04<br>aA#	0.37±00.03<br>aAB#	0.37±0.03<br>aAB#	0.21±0.00<br>abA#	0.26±0.04<br>aB#	nd<br>bAB#
937	943	6.31	**α-Pinene**	3.95±0.01<br>a A #	5.60±1.49<br>b A #	4.79±1.05<br>b A #	4.12±0.56<br>a A #	5.19±1.63<br>b A #	5.92±0.74<br>b A #	3.23±0.15<br>a A #	6.85±0.23<br>b A #	6.53±0.85<br>b A #	4.00±0.76<br>a A #	10.00±0.47<br>b A #	5.37±0.31<br>a A #
955	956	6.71	**Camphene**	2.12±0.87<br>aA#	3.52±0.93<br>aA#	2.55±0.88<br>aA#	2.24±0.79<br>aA#	3.44±1.08<br>aA#	3.56±0.46<br>aA#	2.03±0.06<br>aA#	4.55±0.15<br>bA#	4.03±0.46<br>abA#	2.52±0.50<br>aA#	5.94±0.28<br>aA#	2.91±0.13<br>aA#
979	982	7.5	**β-Pinene**	1.04±0.46<br>aA#	1.47±0.46<br>aA#	1.37±0.48<br>aA#	0.97±0.42<br>aA#	1.34±0.44<br>aA#	1.81±0.18<br>aA#	0.84±0.03<br>aA#	1.82±0.04<br>aA#	1.88±0.33<br>aA#	1.18±0.23<br>abA#	2.12±0.05<br>aA#	0.32±0.01<br>bA#
990	995	7.89	**β-Myrcene**	0.21±0.04<br>aA#	0.30±0.06<br>aAB#	0.24±0.07<br>aA#	nd<br>aA#	0.23±0.07<br>aA#	0.27±0.01<br>aA#	nd<br>aA#	0.29±0.04<br>aAB#	0.23±0.05<br>aA#	0.20±0.00<br>aA#	0.38±0.03<br>abB#	2.13±0.08<br>bA#
1002	1008	8.3	**α-Phellandrene**	0.25±0.04<br>aA#	0.65±0.09<br>bAB#	0.39±0.07<br>abAB#	nd<br>aA#	0.54±0.07<br>bA#	0.38±0.02<br>bAB#	nd<br>aA#	0.71±0.03<br>bAB#	0.56±0.02<br>bB#	nd<br>aA#	1.02±0.01<br>aB#	nd<br>aA#
1024	1027	8.92	**p-Cymene**	0.36±0.07<br>aA#	0.87±0.07<br>bAB#	0.59±0.11<br>abAB#	0.26±0.15<br>aAB#	0.76±0.15<br>abAB#	0.91±0.08<br>bA#	nd<br>aB#	0.69±0.10<br>aB#	0.58±0.05<br>aAB#	0.22±0.04<br>aAB#	1.11±0.11<br>bA#	0.38±0.06<br>abB#
1032	1031	9.05	**Limonene**	**10.18±0.68<br>a A #**	9.06±1.21<br>a A #	8.84±1.42<br>a AB #	7.72±0.75<br>a A #	7.15±1.46<br>a A #	9.89±0.60<br>a AB #	3.52±0.20<br>a A #	7.49±0.86<br>a A #	7.09±1.17<br>a B #	9.61±1.58<br>a A #	**11.89±0.58<br>ab A #**	**13.78±0.29<br>b A #**
1050	1041	9.34	**β-E-Ocimene**	0.32±0.03<br>aA#	0.23±0.02<br>aA#	0.26±0.03<br>aAB#	0.21±0.10<br>aAB#	0.20±0.04<br>aA#	0.21±0.02<br>aAB#	nd<br>aB#	0.23±0.02<br>aA#	nd<br>aB#	0.30±0.02<br>abAB#	0.27±0.01<br>aA#	0.47±0.07<br>bA#
1036	1034	9.13	**1,8-Cineol**	**17.02±0.13<br>a A #**	**15.71±1.98<br>a AB #**	**18.60±1.15<br>a A #**	**17.73±1.45<br>a A #**	**13.96±1.69<br>a A #**	**18.40±1.64<br>a A #**	**16.63±0.93<br>a A #**	**16.68±0.34<br>a AB #**	**19.21±2.84<br>a A #**	**18.33±2.37<br>a A #**	**18.47±1.43<br>a B #**	**22.89±1.75<br>a A #**
1146	1148	12.87	**Camphor**	6.78±0.28<br>a AB #	3.97±0.08<br>a A #	3.82±0.21<br>a A #	4.50±0.98<br>a A #	3.44±0.37<br>a AB #	3.07±0.38<br>a A #	6.94±0.10<br>a B #	2.98±0.11<br>a AB #	3.21±0.30<br>a A #	6.27±0.28<br>a AB #	2.86±0.12<br>b B #	4.23±0.22<br>ab A #
1169	1170	13.59	**Borneol**	3.92±0.38<br>aA#	5.24±0.72<br>aA#	4.12±0.22<br>aA#	3.43±0.47<br>aAB#	4.89±0.44<br>aA#	3.66±0.29<br>aA#	3.46±0.13<br>aAB#	4.82±0.38<br>bA#	4.06±0.19<br>abA#	2.39±0.19<br>abB#	4.45±0.15<br>aA#	2.13±0.06<br>bA#
1177	1181	13.98	**Terpinen-4-ol**	0.31±0.00<br>aA#	0.32±0.06<br>aA#	0.35±0.03<br>aA#	0.33±0.04<br>aA#	0.33±0.06<br>aA#	0.29±0.05<br>aA#	0.40±0.13<br>aA#	0.33±0.04<br>aA#	0.28±0.01<br>aA#	0.28±0.02<br>aA#	0.36±0.03<br>aA#	0.29±0.02<br>aA#
1189	1195	14.43	**α-Terpineol**	0.86±0.11<br>aA#	0.92±0.15<br>aA#	0.83±0.00<br>aA#	0.69±0.06<br>aA#	0.90±0.14<br>aA#	0.74±0.04<br>aA#	0.90±0.03<br>aA#	0.77±0.05<br>aA#	0.73±0.08<br>aA#	0.69±0.03<br>aA#	0.67±0.05<br>aA#	0.51±0.01<br>aA#
1285	1291	17.65	**Bornyl acetate**	8.94±0.42<br>aA#	9.30±0.89<br>aA#	8.63±0.29<br>aA#	8.71±0.39<br>aA#	9.93±1.04<br>aA#	7.40±0.52<br>aA#	5.78±0.20<br>aA#	8.19±0.54<br>bA#	7.54±0.20<br>abA#	5.55±0.18<br>abA#	5.89±0.13<br>aA#	5.03±0.24<br>bA#
1350	1356	19.72	**α-Terpinyl acetate**	2.99±0.38<br>aA#	1.71±0.18<br>bA#	2.36±0.13<br>abA#	3.26±0.31<br>aA#	1.70±0.08<br>bA#	2.23±0.15<br>abA#	3.43±0.26<br>aA#	1.56±0.17<br>bA#	1.97±0.08<br>abA#	2.99±0.27<br>aA#	1.28±0.10<br>bA#	2.64±0.04<br>abA#

1409	1418	21.47	**α-Gurjunene**	0.32±0.06 aA#	0.64±0.09 aA#	0.44±0.03 aA#	0.38±0.08 aA#	0.60±0.09 aA#	0.39±0.05 aA#	nd aA#	0.68±0.06 aA#	0.40±0.07 aA#	nd aA#	0.57±0.01 aA#	nd aA#
1419	1429	21.71	**β-Caryophyllene**	4.64±0.50 ab A #	3.65±0.42 a A #	5.72±0.22 b AB #	5.09±0.40 a A #	4.28±0.50 a A #	5.01±0.28 a AB #	4.94±0.14 a A #	3.86±0.08 b A #	4.62±0.08 ab B #	6.59±0.52 a A #	3.23±0.03 b A #	6.07±0.32 ab A #
1441	1450	22.16	**Aromadendrene**	0.35±0.05 aA#	0.74±0.09 aA#	0.56±0.05 aA#	0.41±0.01 aA#	0.75±0.08 aA#	0.48±0.00 aA#	nd aA#	0.62±0.05 aA#	0.43±0.02 aA#	nd aA#	0.63±0.11 aA#	nd aA#
1454	1464	22.48	**α-Humulene**	6.12±0.60 a A #	4.15±0.52 a A #	6.32±0.19 a AB #	7.14±0.81 a A #	4.56±0.41 b A #	5.46±0.37 ab AB #	6.57±0.19 a A #	4.25±0.17 b A #	4.89±0.12 ab B #	9.26±0.86 a A #	3.63±0.14 b A #	7.28±0.04 ab A #
1461	1472	22.64	**Alloaromadendrene**	6.86±0.79 a AB #	**11.16±1.22 b A #**	8.74±0.34 ab A #	8.59±0.76 ab A #	**11.32±1.32 a A #**	6.79±0.51 b AB #	0.24±0.03 a B #	**9.41±0.45 b A #**	6.43±0.19 ab AB #	0.34±0.14 a AB #	**9.23±0.35** a A #	0.32±0.02 a B #
1478	1525	23.66	**Muurolene**	0.69±0.07 aA#	nd aA#	0.55±0.04 aA#	0.73±0.05 aA#	nd aA#	0.49±0.11 aA#	1.30±0.20 aA#	0.35±0.00 aA#	0.49±0.10 aA#	0.98±0.09 aA#	nd aA#	1.05±0.04 aA#
1590	1587	25.12	**Globulol**	1.24±0.16 aA#	1.51±0.29 aA#	1.15±0.03 aA#	1.32±0.29 aA#	1.56±0.12 aA#	0.98±0.05 aA#	0.58±0.05 aA#	1.29±0.06 aA#	0.90±0.04 aA#	0.43±0.04 aA#	1.08±0.06 aA#	0.24±0.00 aA#
1642	1656	25.67	**τ-Cadinol**	1.83±0.25 a A #	nd aA#	1.09±0.14 a A #	2.05±0.42 a AB #	nd aA#	1.09±0.06 a A #	3.67±0.21 a B #	nd aA#	1.12±0.06 a A #	3.22±0.40 a AB #	nd aA#	2.98±0.02 a A #
**Temperature 30°C**															
**RI lit**	**RI calc**	**RT**	**Name**	**49***	**49****	**49*****	**98***	**98****	**98*****	**147***	**147****	**147*****	**196***	**196****	**196*****
930	937	6.13	**α-Thujene**	0.15±0.00 aA#	0.19±0.03 aA#	0.17±0.01 aA#	0.17±0.01 abAB#	0.14±0.02 aA#	5.72±1.09 bB#	0.24±010 aB#	0.24±0.01 aA#	0.021±0.00 aAB#	0.21±0.02 aAB#	0.23±0.03 aA#	0.24±0.02 aAB#
937	943	6.31	**α-Pinene**	7.03±1.18 a A #	10.23±0.69 b A #	7.90±0.48 b A #	4.79±1.82 a A ##	9.65±1.68 b A #	5.72±1.09 a AB ##	3.54±0.11 a A #	7.11±0.90 b A ##	5.63±0.15 a B ##	5.07±0.23 a A #	6.61±0.20 a A #	6.29±0.03 a AB #
955	956	6.71	**Camphene**	4.04±0.65 aA#	6.18±0.14 aA#	4.05±0.03 aA#	3.43±0.77 aA#	5.94±0.69 aA#	3.26±0.58 aAB#	2.73±0.08 aA#	4.66±0.52 bA#	3.22±0.14 abB#	3.58±0.13 aA#	4.53±0.10 aA#	3.60±0.00 aAB
979	982	7.5	**β-Pinene**	1.62±0.32 aA#	1.84±0.06 aA#	2.45±0.05 aA#	1.78±0.35 aA#	1.54±0.17 aA#	2.23±0.42 aA#	1.70±0.03 aA#	1.64±0.09 aA#	2.51±0.09 aA#	1.97±0.05 abA#	1.56±0.02 aA#	2.72±0.01 bA#
990	995	7.89	**β-Myrcene**	0.31±0.07 aA#	0.37±0.01 aA#	0.45±0.03 aA#	0.49±0.09 aA#	0.51±0.09 aA#	0.45±0.07 aA#	0.27±0.03 aA#	0.38±0.10 abA#	0.51±0.02 bA #	0.42±0.03 aA#	0.44±0.01 aA#	0.69±0.04 aA#
1002	1008	8.3	**α-Phellandrene**	0.16±0.02 aA#	0.80±0.09 aA#	0.17±0.00 aA##	0.19±0.03 aA#	1.09±0.17 aA#	nd aA#	nd aA#	0.89±0.08 aA#	nd aA#	0.19±0.01 aA#	1.01±0.02 aA#	0.18±0.01 aA#
1024	1027	8.92	**p-Cymene**	0.42±0.05 aAB#	0.80±0.09 bA#	0.68±0.03 abAB#	0.51±0.06 aA#	1.13±0.13 bA#	0.59±0.05 abA	0.22±0.02 aB	0.84±0.29 bA#	0.69±0.00 abAB#	0.34±0.02 aAB#	0.78±0.05 aA#	0.74±0.03 aB#
1032	1031	9.05	**Limonene**	**10.50±1.54 ab A #**	8.25±0.62 [a] A #	**11.73±0.86 b AB ##**	**11.81±2.16 a A #**	**10.55±0.17 a A #**	9.73±1.16 a A #	6.81±0.22 [a] A ##	7.63±1.09 ab A #	**11.67±0.41 b AB ##**	**11.77±0.41 ab A ##**	8.25±0.13 a A ##	**14.63±0.21 b B ##**
1050	1041	9.34	**β-E-Ocimene**	0.30±0.03 aA#	nd bA#	0.17±0.01 abA#	0.63±0.03 aA#	0.16±0.00 bA#	0.22±0.01 abAB#	0.32±0.10 aA#	0.13±0.00 bA#	0.23±0.01 abAB#	0.50±0.02 aA#	0.15±0.02 bA#	0.40±0.01 abB#
1036	1034	9.13	**1,8-Cineol**	**13.54±0.83 ab A #**	**10.16±0.45 a A ##**	**15.23±0.43 b A #**	**16.20±0.73 a AB #**	**11.34±0.93 a A ##**	**17.90±0.61 a AB #**	**18.74±0.04 a B ##**	**11.91±0.74 b A ##**	**18.12±0.49 ab B #**	**16.39±0.18 ab AB #**	**12.19±0.19 a A ##**	**17.22±0.21 b AB ##**
1146	1148	12.87	**Camphor**	7.61±0.27 a A ##	5.55±0.06 ab A ##	5.27±0.23 b A ##	9.85±0.71 a A #	7.63±0.09 a B ##	8.02±0.45 a B ##	9.88±0.08 a A ##	7.13±0.18 ab AB ##	6.09±0.17 b AB ##	8.17±0.14 a A #	5.86±0.17 a AB ##	5.73±0.09 a AB ##

1169	1170	13.59	**Borneol**	2.79±0.25 abA#	4.81±0.08 aAB#	2.29±0.10 bA#	3.10±0.32 aAB##	4.83±0.40 aA#	3.36±0.31 aB#	4.44±0.12 abB#	5.77±0.28 aAB#	3.01±0.02 bAB#	3.18±0.11 abAB#	6.00±0.08 aB#	2.63±0.05 bAB#
1177	1181	13.98	**Terpinen-4-ol**	0.43±0.01 aA#	0.40±0.01 aA#	0.43±0.02 aA#	0.46±0.04 aA#	0.46±0.01 aAB##	0.49±0.03 aA#	0.47±0.01 aA#	0.51±0.04 aAB##	0.46±0.02 aA#	0.46±0.02 abA#	0.54±0.03 aB#	0.42±0.01 bA#
1189	1195	14.43	**α-Terpineol**	0.91±0.08 aA#	0.86±0.04 aA#	0.76±0.02 aA#	0.98±0.13 aA#	1.03±0.12 aA#	0.90±0.08 aA#	1.10±0.01 aA#	1.18±0.03 aA#	0.82±0.01 aA#	1.03±0.02 aA#	1.12±0.00 aA#	0.84±0.04 aA#
1285	1291	17.65	**Bornyl acetate**	8.59±0.71 abA#	10.26±0.35 aA#	7.91±0.34 bA#	8.48±0.71 aA#	9.81±0.61 aA#	8.46±0.54 aA#	8.40±0.09 abA#	10.28±1.08 aA#	8.18±0.19 bA#	8.23±0.12 abA#	10.85±0.13 aA#	7.73±0.12 bA#
1350	1356	19.72	**α-Terpinyl acetate**	3.88±0.32 aA#	1.70±0.02 aA#	3.77±0.22 aA#	3.91±0.41 aA#	1.54±0.19 aA#	3.79±0.32 aA#	4.13±0.04 aA#	1.78±0.12 bA#	3.79±0.05 abA#	3.95±0.06 aA#	1.77±0.03 bA#	3.63±0.02 abA#
1409	1418	21.47	**α-Gurjunene**	nd aA#	0.85±0.06 bB#	nd aA#	nd aA#	0.85±0.01	nd aA#	nd aA#	0.93±0.14 aA#	nd aA#	nd aA#	0.93±0.03 aA#	nd aA#
1419	1429	21.71	**β-Caryophyllene**	7.45±0.65 ab A ##	3.83±0.25 a A #	8.46±0.50 b A ##	7.73±1.02 a A #	3.69±0.43 a A #	7.47±0.64 a A ##	7.90±0.11 ab A ##	4.73±0.69 a A ##	8.46±0.25 b A ##	8.30±0.13 ab A ##	5.00±0.06 a A ##	8.58±0.15 b A ##
1441	1450	22.16	**Aromadendrene**	nd aA#	1.38±0.10 bB#	nd aA#	nd aA#	1.45±0.17	nd aA#	nd aA#	1.62±0.18	nd aA#	nd aA#	1.63±0.05	nd aA#
1454	1464	22.48	**α-Humulene**	8.84±0.90 a A ##	4.19±0.31 a A #	8.73±0.56 a A ##	8.38±1.14 a A #	3.89±0.49 a A #	7.47±0.55 a A ##	8.58±0.16 a A ##	4.68±0.51 b A #	8.19±0.31 ab A ##	8.60±0.04 a A #	4.66±0.08 b A ##	8.08±0.17 ab A #
1461	1472	22.64	**Alloaromadendrene**	0.33±0.04 ab AB ##	**11.08±0.67 a A #**	0.25±0.02 b A ##	0.22±0.10 a A ##	**10.37±1.37 a A #**	0.28±0.00 a A ##	0.44±0.02 ab B ##	**11.86±1.03 a A ##**	0.43±0.01 b A #	0.36±0.05 ab AB #	**11.53±0.10 a A #**	0.30±0.03 b A #
1478	1525	23.66	**γ-Muurolene**	1.73±0.37 aA#	0.43±0.19 bB#	1.74±0.10 aA#	1.56±0.16 aA#	0.29±0.13 bB#	2.06±0.21 aA#	1.98±0.03 aA#	0.22±0.03 bB#	2.07±0.04 aA#	1.62±0.02 aA#	0.17±0.00 bB#	1.69±0.07 aA#
1590	1587	25.12	**Globulol**	0.57±0.05 aA#	1.86±0.09 bB#	0.34±0.04 aA#	0.37±0.05 aA#	1.65±0.18 bB#	0.29±0.01 aA#	0.27±0.01 aA#	1.82±0.11 bB#	0.20±0.00 aA#	nd aA#	1.91±0.02 bB#	nd aA#
1642	1656	25.67	**τ-Cadinol**	5.25±0.77 a A##	0.14±00 b A #	5.62±0.42 a AB ##	5.40±0.67 a A #	nd bB#	6.79±0.42 a A ##	6.52±0.09 a A ##	0.18±0.01 b A ##	6.36±0.22 a AB ##	4.74±0.09 a A ##	nd bB#	4.98±0.12 a B ##

Table 3S. Relative contribution of light intensity (LI), temperature (T) and culture duration (CD) and their interactions to variation in the morphogenetic response of *S. yangii in vitro* shoots. Statistical significance : not statistically significant (n.s.) $p>0.05$; *$p\leq0.05$.

Source of variation	**Mean number of axillary shoots per explant**	**Explants developing two axillary shoots**	**Mean length of axillary shoots [cm]**	**Mean number of nodes/cm of axillary shoot**	**Mean mass of shoot [g]**	**Mean roots length [cm]**
LI	18.074	14.710	53.183	80.610	42.624	3.628
T	56.394	51.650	18.723	3.314	22.740	0.793
CD	9.469	5.726	0.887	0.186	5.900	79.591
LI × T	0.665	2.448	13.752	7.260	5.550	4.936
LI× CD	8.337	12.334	4.102	2.770	10.660	5.969
T × CD	3.551	7.020	4.806	0.416	8.810	4.067
LI × T × CD	2.001	2.363	3.077	2.585	2.410	0.776
	**p - values**					
LI	0.000*	0.021*	0.000*	0.000*	0.000*	0.000*
T	0.000*	0.000*	0.000*	0.282	0.000*	0.072
CD	0.000*	0.207	0.613	0.978	0.004*	0.000*
LI × T	0.644	0.521	0.000*	0.080	0.015*	0.000*
LI× CD	0.000*	0.004*	0.011*	0.446	0.000*	0.000*
T × CD	0.072	0.134	0.021*	0.932	0.000*	0.000*
LI × T × CD	0.244	0.706	0.053	0.492	0.090	0.006*

Table 4S. Relative contribution of light intensity (LI), temperature (T) and culture duration (CD) and their interactions to variation in non-VOCs and VOCs content of *S. yangii in vitro* shoots. Statistical significance : not statistically significant (n.s.) p>0.05; *p≤0.05.

Source of variation	**Non-VOCs**		**VOCs**							
	RA	CA	α-Pinene	Limonene	1.8-Cineole	Camphor	β-Caryophyllene	α-Humulene	Alloaromadendrene	τ-Cadinol
LI	33.790	14.504	53.919	25.648	36.066	22.567	40.852	68.774	66.376	46.634
T	0.435	13.695	22.085	20.950	34.420	70.138	47.136	17.476	15.612	39.209
CD	10.531	20.009	2.408	32.458	13.644	0.601	2.366	2.892	3.638	0.683
LI × T	34.175	5.527	0.540	9.623	8.891	0.709	6.467	4.728	7.620	11.187
LI× CD	2.745	0.643	0.899	3.478	1.221	1.619	0.852	1.460	1.061	0.611
T × CD	15.820	43.031	14.703	3.790	3.270	3.856	1.440	2.756	4.804	1.083
LI × T × CD	2.431	2.492	4.749	2.906	0.982	0.240	0.543	1.444	0.843	0.504
**p - values**										
LI	0.000*	0.000*	0.000*	0.000*	0.000*	0.000*	0.000*	0.000*	0.000*	0.000*
T	0.018*	0.000*	0.000*	0.000*	0.000*	0.000*	0.000*	0.000*	0.000*	0.000*
CD	0.000*	0.000*	0.023*	0.000*	0.000*	0.097	0.001*	0.001*	0.000*	0.000*
LI × T	0.000*	0.000*	0.467	0.001*	0.005*	0.083	0.000*	0.000*	0.000*	0.000*
LI× CD	0.000*	0.000*	0.280	0.013*	0.566	0.000*	0.036*	0.012*	0.000*	0.000*
T × CD	0.000*	0.000*	0.000*	0.028*	0.103	0.000*	0.010*	0.002*	0.000*	0.000*
LI × T × CD	0.000*	0.000*	0.000*	0.032*	0.688	0.509	0.174	0.013*	0.000*	0.000*

Table 5S. Relative contribution of light intensity (LI), temperature (T) and culture duration (days) and their interactions to variation in chlorophyll, DPPH, TPC and TBARS content of *S. yangii in vitro* shoots. Statistical significance : not statistically significant (n.s.) p>0.05; *p≤0.05.

Source of variation	**Chl a**	**Chl b**	**Car**	**Chl a/Chl b**	**(Chl a+Chl b)/ Car**	**TBARS**	**TPC**	**DPPH**
LI	40.308	46.413	36.700	2.397	52.697	35.032	67.962	7.568
T	0.833	4.739	0.216	78.189	0.000	13.528	3.561	37.175
CD	2.995	3.427	4.142	3.041	1.940	6.196	11.848	16.393
LI × T	26.947	17.080	24.181	4.123	14.143	4.747	6.288	6.231
LI× CD	15.293	17.333	19.139	6.912	7.265	0.375	3.259	6.120
T × CD	4.671	1.345	8.945	3.815	7.398	36.792	5.826	18.379
LI × T × CD	8.835	9.508	6.485	1.405	16.249	3.098	1.240	6.319
**p – values**								
LI	0.000*	0.000*	0.000*	0.000*	0.000*	0.000*	0.000*	0.020*
T	0.008*	0.000*	0.290	0.000*	0.981	0.000*	0.000*	0.000*
CD	0.000*	0.000*	0.000*	0.000*	0.000*	0.000*	0.000*	0.000*
LI × T	0.000*	0.000*	0.000*	0.000*	0.000*	0.000*	0.000*	0.038*
LI× CD	0.000*	0.000*	0.000*	0.000*	0.000*	0.146	0.000*	0.006*
T × CD	0.000*	0.000*	0.000*	0.000*	0.000*	0.000*	0.000*	0.000*
LI × T × CD	0.000*	0.000*	0.000*	0.000*	0.000*	0.000*	0.000*	0.005*
